# Swimming Warm-Up and Beyond: Dryland Protocols and Their Related Mechanisms—A Scoping Review

**DOI:** 10.1186/s40798-022-00514-y

**Published:** 2022-09-24

**Authors:** Francisco Cuenca-Fernández, Daniel Boullosa, Óscar López-Belmonte, Ana Gay, Jesús Juan Ruiz-Navarro, Raúl Arellano

**Affiliations:** 1 Aquatics Lab. Department of Physical Education and Sports. Faculty of Sport Sciences, University of Granada, Ctra. Alfacar SN (18071), Granada, Spain; 2 International Strength and Conditioning Society, Murcia, Spain; 3 Integrated Institute of Health, Federal University of Mato Grosso Do Sul, Campo Grande, Brazil; 4 College of Healthcare Sciences, James Cook University, Townsville, Australia; 5 Research and Development Department, iLOAD Solutions, Campo Grande, Brazil

**Keywords:** Priming strategies, Acute exercise, PAPE, Competition preparation, Conditioning, Water sports

## Abstract

In swimming, the beneficial effects of the in-water warm-up are often undermined by the long transition periods before competition (≥ 20 min). For that reason, studies comparing the effects of in-water warm-ups followed by dryland activities have been conducted in the swimming literature. This has brought conflicting evidence due to large combinations of supervised and unsupervised warm-up procedures used. Therefore, a scoping review was performed to discuss (1) why warm-up strategies are important for competitive swimming; to identify (2) what are the different warm-up approaches available in the literature, and; to establish (3) what are the main conclusions, considerations and gaps that should be addressed in further research to provide clearer guidance for interventions. The search was conducted on PubMed, Web of Science, Scopus, and SPORTDiscus databases. To be considered eligible, studies must have assessed acute short-term responses of warm-up procedures in swimmers by using randomized controlled trials or pre-post study designs. A total of 42 articles were included in this review. The effectiveness of warm-up responses was evaluated based on the inclusion or not of warm-up, the type of conditioning activity (in-water exercise, in-water exercise combined with dryland or dryland exercise only), its duration, and intensity. (1) Warm-up mechanisms have been mainly related to temperature changes associated to cardiovascular adaptations and short-term specific neuromuscular adaptations. Thus, maintaining muscle activity and body temperature during the transition phase immediately prior to competition could help swimmers' performance; (2) the most common approach before a race usually included a moderate mileage of in-water warm-up (~ 1000 m) performed at an intensity of ≤ 60% of the maximal oxygen consumption, followed by dryland protocols to keep the muscle activity and body temperature raised during the transition phase. Dryland activities could only optimize performance in sprint swimming if performed after the in-water warm-up, especially if heated clothing elements are worn. Using tethered swimming and hand-paddles during warm-ups does not provide superior muscular responses to those achieved by traditional in-water warm-ups, possibly because of acute alterations in swimming technique. In contrast, semi-tethered resisted swimming may be considered as an appropriate stimulus to generate post-activation performance enhancements; (3) nothing has yet been investigated in backstroke, butterfly or individual medley, and there is a paucity of research on the effects of experimental warm-ups over distances greater than 100 m. Women are very under-represented in warm-up research, which prevents conclusions about possible sex-regulated effects on specific responses to the warm-up procedures.

**Key Points**
1. An in-water warm-up of 1,000-1,200m followed by various sets of full-body ballistic conditioning activities such as med ball throws, resisted bands, and explosive jumps for no more than 5 min, and with no more than 10-15 min of transition phase, could be effective in improving swimming performance.
2. It is possible that additional specific neuromuscular adaptations may occur during warm-ups conducted in the water, which is why their implementation or combination with dryland activities is recommended.
3. Certain subgroups of athletes may obtain positive effects, while others may see their performance impaired from the same intervention. Thus, it seems important to examine the role of intra-individual response variations to a given stimulus.

## Introduction

Warm-up activities are used to enhance subsequent exercise [1–3]. The current techniques can be classified into two broad categories: passive or active warm-up. Passive warm-up involves increasing muscle or core temperature without depleting energy substrates by some external means, such as hot showers, baths, saunas, diathermy, and heating pads [1, 4]. Although changes in performance can be mostly attributed to temperature-related mechanisms [5], these methods are mainly used as practices to maintain body temperature between warm-up and the task [6]. In the active warm-up, on the contrary, the muscle temperature is raised by means of body exercises, such as running, cycling or calisthenics [2, 4, 7], as these activities have been demonstrated to optimize muscle glycolysis and phosphate degradation during subsequent exercise [8]. In fact, it has not been demonstrated that the temperature increases are solely capable of increasing blood flow to accelerate oxygen consumption ($\dot{\text{V}}{\mathrm{O}}_{2}$) [1], thus decreasing the reliance on anaerobic metabolism at the beginning of exercise [9, 10]. Therefore, the oxygen supply to muscles could be affected by additional metabolic responses that occur only during active warm-up.

For instance, increased hydrogen ion (H +) concentration during muscle contraction has been reported to cause vasodilation and increased muscle blood flow [11], which helps to pump blood from the heart to all parts of the body more easily and rise body temperature [2, 5]. Indeed, some studies have determined that at least 10–15 min of exercise at 70% of maximal heart rate (HR) is necessary to induce a 1–2 ºC rise in body temperature [12], with a 4–10% improvement in peak power output for every 1 °C increase [13]. Therefore, it is possible that an acceleration of the overall $\dot{\text{V}}{\mathrm{O}}_{2}$ kinetics is due to the increased oxygen supply obtained with augmented blood flow to the muscles which, in turn, could be associated with an increase in body temperature [10, 14, 15]. The warm-up activities also increase the nerve conduction rate by increasing the speed of nerve impulse transmission [16]. In addition, these activities have additional muscle mechanical effects, such as a decrease in muscle stiffness by "breaking" the stable bonds between actin and myosin filaments [17], which would decrease viscous resistance of muscles and joints [18].

In view of these aspects, the athletic community has traditionally shown a considerable interest in the performance enhancements seen soon after warm-up [4]. Specifically, it has been reported that 5–10 min active warm-up of moderate intensity could significantly improve short-term performance on a range of tasks [1, 2]. Cardiovascular and neuromuscular factors triggered by the warm-up typically appear with a delay of ~ 3–5 min and last, at least, 5–10 min [19, 20]. However, even though very intense exercise could deplete energy reserves generating acute fatigue [21–23], this is significantly reduced during the first few minutes of recovery [1, 19, 20]. Therefore, this provides a "window of opportunity" after the fatigue generated by the warm-up, which is maintained for a period during which the athlete may be able to benefit from an ergogenic advantage from the enhanced state. This effect has been identified in the literature as post-activation performance enhancement (PAPE) [24], and should be considered one of the goals prompted by voluntary tasks after specific warm-ups [4].

The course of any performance enhancement is individually regulated depending on the type, duration and intensity of the conditioning activity (CA) [25, 26]; the participant's background and the recovery interval provided [20, 26], and the performance parameter to be assessed, including the verification test chosen [27]. Research has supported the positive effects of high-loaded exercises on subsequent explosive movements based on the fact that, an augmented stiffness and fiber recruitment may be useful in regulating force output during stretch–shortening cycle movements [28–30]. However, this strategy seems to be more effective for speed-power athletes (e.g., sprinters and jumpers), while endurance athletes (e.g., marathon runners and triathletes) would probably obtain performance enhancements from submaximal prolonged conditioning activities due to an optimized balance between fatigue and potentiation [31]. Furthermore, recent PAPE research has also shown the effectiveness of various potentiation protocols for the upper body involving submaximal activities such as resisted bands, or ballistic exercises such as throws, plyometrics and swings [32]. Thus, these features could be relevant for a sport as swimming, as the use of very heavy resistance exercises for the upper body may not be as effective to potentiate as for the lower body, possibly due to the lower muscle mass involved and different muscle fiber composition [33, 34].

At this point, a review of the existing literature could help to provide further evidence on the effectiveness of the different warm-up methods that have been used in swimming. A previous review conducted by Neiva et al. [6], showed that the in-water warm-up had a positive effect on swimmers’ performance, especially for distances longer than 200 m. Thus, they recommended that swimmers should warm up for a distance of 1000 to 1500 m, including short, intensive and specific tasks in some parts of the warm-up at an intensity similar to race pace, and that sufficient recovery time should be provided to avoid the early onset of fatigue and to allow for the restoration of energy reserves (8 to 20 min). At that time, studies on the effects of warm-up were scarce, so these results were truly valuable and set the strategy to be followed in current interventions. However, due to the growing interest in the topic, a large number of studies have been published in recent years incorporating other methods in addition to in-water routines, with an increasing trend toward the use of dryland exercises and/or different combinations with other means such as heated clothing and resistive equipment. Therefore, the aim of this scoping review was to compare the effects of supervised and unsupervised warm-up protocols on competitive swimming performances to update the current knowledge and provide clearer guidance for interventions.

## Methods

### Search Strategy

A literature search was performed in accordance with the guidelines provided for scoping reviews [35]. This approach, based on the work of Arksey and O’Malley [35], considered research findings and reached conclusions from the existing literature in relation to the state of research activity in warm up techniques for swimmers, with the aim of quickly mapping the key concepts underpinning an area of research in fields that incorporate a wide range of study designs [36–38]. This type of scoping review facilitates also the identification of gaps in the evidence base where no research has previously been conducted [39]. The focus of our review identified the following research questions:

- 1. Why warm-up strategies are important for competitive swimming?
- 2. What are the different warm-up approaches available in the literature?
- 3. What are the main conclusions, considerations and gaps that should be addressed in further research?

The identification of relevant studies was conducted encompassing publications from inception to 2nd November 2021 on four international electronic databases: PubMed, Web of Science (all collections), Scopus, and SPORTDiscus. The literature search was performed in accordance with the guidelines provided in the Preferred Reporting Items for Systematic Review and Meta-Analyses, with the extension for Scoping Reviews (PRISMA – ScR) [39]. Boolean operators were applied to search the article title, abstract and keywords as follow: (((((((((((post-activation potentiation) OR (PAP)) OR (warm-up)) OR (resisted warm-up)) OR (pre-race)) OR (post-activation performance enhancement)) OR (PAPE)) OR (preparedness)) OR (pre-activation)) OR (acute)) AND (swimming)) OR (start performance)) AND (swimmers). Specificities of each search engine: i) in PubMed, the search was limited to title or abstract; publications were limited to randomized controlled trials, excluding books and documents, meta-analyses, reviews and systematic reviews; ii) in Web of Science, “topic” was the term used to refer to title, abstract and keywords; iii) in Scopus, the publication type was limited to article, and; iv) in SPORTDiscus, the search was limited to articles in peer-reviewed journals. An update of the database search was conducted from November 2021 to June 2022, following the same steps as during the original search.

### Eligibility Criteria

Inclusion criteria were defined as follows: i) randomized controlled trial or pre-post study design; ii) competitive swimmers ≥ 13 years old, with at least three years of competitive experience; iii) studies that measured acute short-term responses of warm-up procedures on swimming performance; iv) studies that verified the warm-up effects on swimming performance (e.g., swimming start, underwater phase, kinetic or kinematic variables of swimming).

Exclusion criteria were: i) non-swimmers (i.e., water polo players, triathletes, scuba divers) or animals; ii) swimming performance was not measured; iii) studies conducted exclusively in dry-land settings, with no transference to any swimming component; iv) studies including dietary supplements; v) reviews, case-studies, poster, conference abstracts, or presentations; vi) not written in English.

### Data Analysis

The effectiveness of warm-up responses was evaluated based on the known factors that influence subsequent performance: the inclusion or not of warm-up, the type of CA (including duration and intensity), and the rest provided, both for control and experimental interventions [23, 40]. This assessment included kinematic measures, such as time, distance and speed, including the stroke patterns (e.g., stroke length and stroke rate); kinetic measures such as force, power, impulse or rate of force development (RFD); and physiological measures such as lactate, temperature, heart rate, oxygen saturation, hemoglobin concentration and rate of perceived effort (RPE). In this scoping review, swimming times performances were used as the primary outcome reflecting PAPE responses and, irrespective of the statistical significance achieved, relative changes in performance (∆%) were calculated as the percentage difference between conditions (% = [(Mean^b^ – Mean^a^)/Mean^ab^] × 100 [41].

## Results and Discussion

**Fig. 1:**
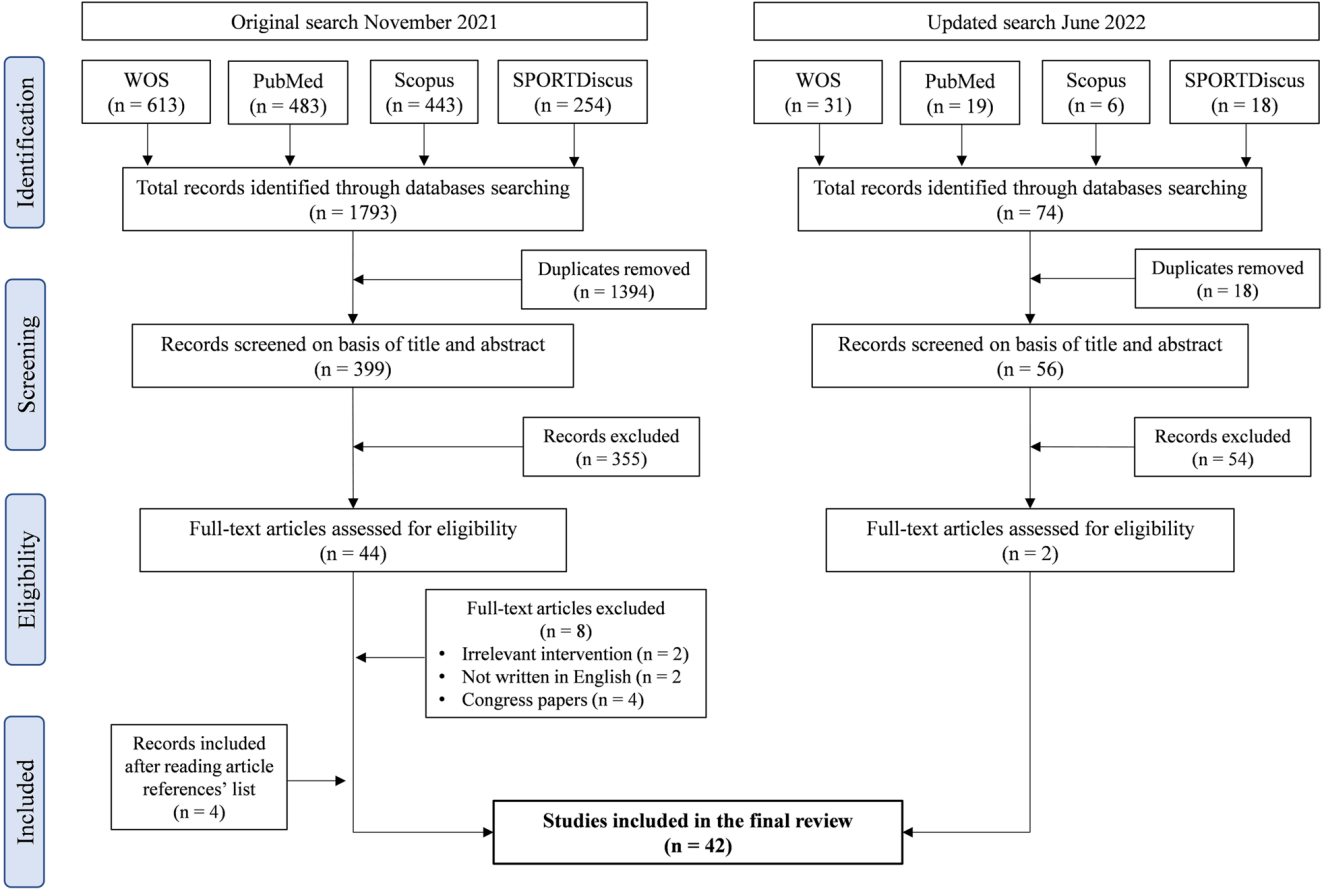
PRISMA – ScR flow diagram for article selection

Initial search returned 1793 results. After removal of duplicates, 399 records remained. Screening the titles and abstracts for eligibility criteria resulted in the exclusion of 355 articles that did not match the eligibility criteria. Following full-text analysis, 36 articles were eligible, to which four additional records were added after reading the reference list of those articles. The updated searches in June 2022 resulted in a total of 74 new articles, of which two new studies were eligible. Therefore, 42 studies were finally included in this review (Fig. 1).

### Why are Warm-up Strategies Important for Competitive Swimming?

The in-water warm-up is a common practice to improve the physiological, psychological and technical preparation of swimmers [6, 40]. Its positive effect was first presented by De Vries [12], a pioneer in testing different modes of warm-up (e.g., in-water, calisthenics, hot showers and massage), with the best effects driven by the in-water warm-up. The main effects of the in-water warm-up could be due to the increase in body temperature and blood flow and oxygen supply to the muscles after exercise [6], although some authors have also pointed out its effects on joint mobility and recalibration of the athletes' sensorimotor network [42, 43], as well as in the reduction of excessive anxiety before competition by familiarizing athletes with the competition venue [44–46].

#### The Transition Phase Between the Warm-up and the Race

At local and regional championships, a swimming event consisting of several races can take up several hours between warm-up and competition [46]. However, after 15–20 min of passive rest, muscle temperature can rapidly decrease and performance can be negatively affected [1]. Although this problem is solved during major swimming events where there is usually a second pool available for swimmers to warm-up, the rules of the International Swimming Federation (FINA) dictate that swimmers must enter the call room at least 20 min before the race to be inspected by technical officials (www.fina.org). This rule therefore poses a problem for swimmers in terms of the time between the completion of their warm-up and the race that can mitigate the positive effects of the warm-up, jeopardizing swimmers' performance. Indeed, other issues in the competitive environment, such as delays in the competition schedule or the time needed to change swimming costumes, may result in even longer transition periods, which may negatively affect performance [15, 47], as has been demonstrated in other exercises and sports [20, 48]. In this regard, some approaches have revealed that shorter duration of transition phases (10–20 min) improved 200 m front crawl performance by 1.38 and 1.48%, respectively, compared to 45 min [49], while a 10 vs 20 min transition phase led to performance increases of 1.12% in 100 m front crawl [50]. Therefore, this suggests that alternative forms of rewarming are required to maintain performance during the transition phase in competitive swimming.

#### The Importance of Maintaining the Warm-up Effects

During international swimming events, marginal differences of < 0.5% separate medal and non-medal positions [51, 52]. For example, for the Rio 2016 Olympic Games, just one hundredth of a second determined the difference between the first and the second-place finisher in the men's 50 m freestyle (www.fina.org). At this level of performance, it cannot be ruled out that swimmers need to maintain an activated muscle system to compete at their best of their ability [53]. For that reason, it is common to see swimmers trying to minimize the negative effects of the waiting period by staying active (e.g., through ballistic stretching, jumping sets, or by punching hard on the chest and limbs) [47, 54, 55]. Therefore, while a good warm-up strategy is crucial, the development of methods to maintain a high muscle temperature and activation during the recovery period emerges as a factor to be considered [13].

Active warm-up activities during the transition phase, such as dryland exercises performed alone or in combination with other passive strategies such as heated tracksuit jackets, have increased their popularity among elite swimming coaches as an alternative tool to be used between the classical in-water warm-up and competition [46, 56–59]. In general, short-term performance is likely to improve if the recovery interval after the CA allows phosphocreatine (PCr) stores to be fully restored [2]. This process is almost completed within the first three min of rest in trained athletes [60, 61], and is facilitated by higher intra-muscle oxygen delivery [62]. However, although a longer recovery period may be necessary for complete resynthesis of PCr [63], such durations could be accompanied by a significant drop in muscle temperature [1]. Therefore, for future interventions it would be necessary to clarify exactly what kind of warm-up and rest strategies offer the best results for effective PAPE effects in swimmers.

### What are the Different Warm-up Approaches Available in the Literature?

**Fig. 2:**
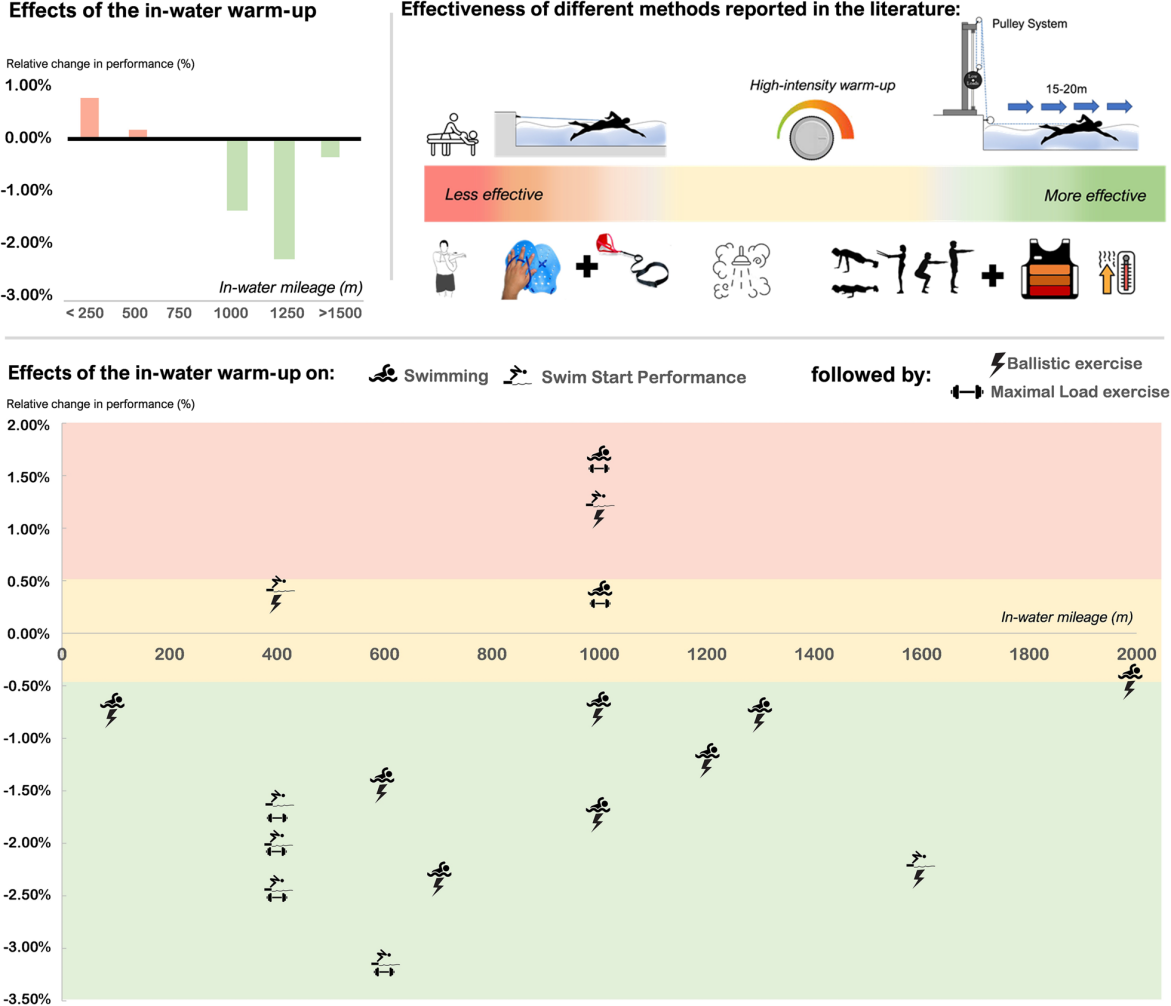
Schema of the effectiveness of the warm-up protocols used in swimming

The literature search yielded multiple combinations that can be gathered in two main groups: i) only in-water warm-up, and; ii) in-water warm-up combined with dryland activities. However, given that warm-up in swimming involves a large number of variables that interact with each other [6], other approaches such as resistive elements added to the in-water warm-up or the realization of dryland warm-ups including heat source equipment without the in-water component, were also gathered and discussed. All these combinations and changes in swimming performance (∆%) are represented in Fig. 2.

#### Only in-Water

##### In-Water Warm-up

Nine studies have explored the differences between warming and a control condition in the swimming literature, obtaining different outcomes (Table 1). First, Mitchell and Huston [64] found no difference in 200-yards time after no warm-up compared to 400-yards warm-up at 70% of $\dot{\text{V}}{\mathrm{O}}_{2\mathrm{max}}$. Subsequently, Romney and Nethery [65], obtained a reduction in 100-m performance three min after a warm-up of superior volume (~ 1000 m), while Bobo [66] did not obtain differences in the average time of a 5 × 100-yards set after a 800-yards warm-up compared to no warm-up. Thus, it seems that the warming effect was more evident in the shorter efforts. In line with this, Neiva et al. [67] obtained superior force values in 30-s tethered swim attached to a load-cell 10 min after 1,000-m warm-up compared to no warm-up, while Balilionis et al. [21] obtained slight improvement in 50-yard time in participants who performed a regular warm-up (~ 1,200 m), compared to no warm-up. In this case, as swimmers perceived the warm-up as "somewhat hard" on the rate of perceived exertion effort (RPE) scale, the results could have been better if more resting time or a lower intensity would have been provided [23]. Adams and Psycharakis [5] obtained only trends for improvements after two warm-ups (10 or 20 min swimming) compared to no warm-up, probably because of the long recovery period (20 min) used. Neiva et al. [68] found that 100-m performance was faster 10 min after the warm-up condition (1000 m), although three swimmers swam faster without any warm-up, also highlighting the highly variable responses between individuals to pre-competitive procedures and fatigue dissipation [25]. Therefore, despite the different responses observed, it could be concluded that the inclusion of an in-water warm-up for the 50–100-m races could lead to performance improvements compared to no warm-up if adequate warm up intensity and rest time are provided between the warm-up and the race.

**Table 1:** In-water warm-up compared to no activity (*n* = 9)

Reference	Participants, Sex & Age	Level & Experience	Control condition	Rest	Experimental condition	Rest	Main findings & results
Adams & Psycharakis [5]	8 males (20.1 ± 1.8 y)	Competitive swimmers	i) 20 min in-water warm-up including a freestyle base set, speciality stroke (containing kick, pull and drills), start and turns, before finishing with a 200 m swim down	20 min	ii) No warm-up: sit in a sauna for 20 min	20 min	The WU condition was most effective on 100-m individual’s preferred stroke; T100m: i: 61.1 ± 6.4 s; ii: 62.2 ± 5.7 s. The HR and RPE were lower after the active warm-up
Balilionis et al. [21]	8 males (19.9 ± 0.6 y) 8 females (19.8 ± 0.7 y)	National collegiate; > 5 years of competitive experience	i) No WU	3 min	ii) Short WU: 50-yards freestyle swim at 40% of their maximal effort and another 50 yards swim at 90% of their maximal effort; iii) Regular WU; their own precompetitive swim WU (males: 1,257 ± 160 m) (females: 1,314 ± 109 m)	3 min	There was large individual variability, and just 44% of the swimmers achieved their fastest time after regular WU. HR was higher before the 50-yard trial after the regular WU; T50 yard: i: 25.19 ± 1.54 s; ii: 25.26 ± 1.61 s iii: 24.95 ± 1.53 s. There was no difference between i and ii. HR: i: 150 ± 19 bpm; ii: 142 ± 16 bpm; iii: 156 ± 23 bpm). RPE: i: 6.3 ± 0.5; ii: 10.1 ± 1.7; iii: 12.1 ± 1.0. There were no differences for the swimming start variables
Bobo et al. [66]	10 males	Highly trained	i) No WU	5 min	ii) In-water exercises (800-yards)	5 min	No differences between conditions in a set of 5 × 100-m freestyle
Houmard et al. [69]	8 males	Highly trained collegiate	i) No WU	5 min	ii) 1500-yards at 65%$\dot{\text{V}}{\mathrm{O}}_{2\mathrm{max}}$; iii) 1300-yards at 65%$\dot{\text{V}}{\mathrm{O}}_{2\mathrm{max}}$+ 4 × 50-yards (110%$\dot{\text{V}}{\mathrm{O}}_{2\mathrm{max}}$)	5 min	Although swimming time performance in 200-yards was not tested, they obtained higher distance per stroke (3.76%) in the longest warm-ups
Mitchell & Huston [64]	10 males (19.3 ± 0.7 y)	Collegiate	i) No WU	5 min	ii) 366-m swim at 70%$\dot{\text{V}}{\mathrm{O}}_{2\mathrm{max}}$(LWU) iii) 4 × 46-m swims at 1 min intervals at a speed corresponding to 110%$\dot{\text{V}}{\mathrm{O}}_{2\mathrm{max}}$(HWU)	5 min	Performance times in the tethered swim were slightly better in the WU conditions; Tethered swim: i: 116.8 ± 46.8 s; ii: 137 ± 53.3 s; iii: 122.94 ± 37.2 s. Lactate: i: 1.73 ± 0.61 mM/L; ii 2.27 ± 0.81 mM/L; iii: 6.97 + 1.97 mM/L
Neiva et al. [68]	10 males (16.0 ± 0.6 y) 10 females (16.2 ± 1.1 y)	Competitive (64.71 s in 100 m, 456 ± 85 FINA points); 7.1 ± 1.2 years of experience and a training frequency of 16.0 ± 1.5 h/week	i) 1,000-m in-water WU (300 m easy swim; 2 × 100 m/15 s rest second faster, hdp; 8 × 50 rest 1 min [25 m kick/25 m complete; 25 m drills/25 m complete; 25 m race pace/25 m easy; 25 m race pace/25 m easy]; 100 m easy swim)	10 min	ii) No WU	10 min	Swimmers were faster in the first 50-m lap of the WU trial, which led to an improvement in overall 100-m performance; different biomechanical patterns were observed after WU or NWU T100m: i: 67.15 ± 5.60 s; ii: 68.10 ± 5.14 s (d = 0.69); T1st50m: i: 32.10 ± 2.59 s; ii: 32.78 ± 2.33 s (d = 0.89); T2nd50m: i: 35.00 ± 3.27 s; ii: 35.37 ± 2.98 s (d = 0.44); RPE did not change; SR: i: 0.77 ± 0.60 Hz; ii: 0.72 ± 0.06 Hz (d = 1.09); SL: i: 1.90 ± 0.18 m; ii: 1.99 ± 0.18 m (d = 0.66)
Neiva et al. [67]	10 males (15.3 ± 0.9 years)	National; 7.2 ± 1.1 years, training for 6 to 9 times per week	i) No WU	10 min	ii) 1,000-m in-water (typical WU frequently performed before a competitive swimming)	10 min	WU improved the maximum and mean propelling forces of the swimmer in front crawl swimming technique. Lactate and RPE remained unvaried; Maximum force: i: 299.62 ± 77.56 N; ii: 351.33 ± 81.85 N; mean force: i: 91.65 ± 14.70 N; ii: 103.97 ± 19.11 N); lactate: (i: 6.27 ± 2.36 mM/L; ii: 6.18 ± 2.32 mM/L; RPE: i: 15.90 ± 2.42; ii: 15.60 ± 2.27
Romney & Nethery, [65]	10 males	Collegiate	i) No WU	20 min	ii) 1,000-m in-water (15 min)	20 min	The swimming warm-up was more effective to improve 100-m freestyle performance than doing nothing T100-yard: ii: -0.75 s; RPE and stroke count did not change

*y* Years old, *WU* Warm-up, *hdp* High distance per stroke, T50–100 m: Time performed in 50–100 m swimming, *d* = Cohen’s d (effect size), *SR*: Stroke rate, *SL* Stroke length, *RPE* Rate of perceived exertion effort

#### In-Water Warm-ups With Different Volume

For swimmers competing several times during the same session, performing long warm-ups may lead to accumulation of higher levels of fatigue that may affect performance [69]. Therefore, if short warm-ups provide similar acute adaptations as long warm-ups, this could be an advantage in terms of preserving energy for subsequent efforts (Table 2). This hypothesis was initially tested by Houmard [69] who tested several warm-ups of different volumes (i: 200-yards; ii: 1500- yards) and, although they did not measure time performances, a larger stroke length was observed after the longest warm-up, which could reflect an increase of swimming efficiency. Subsequently, Balilionis et al. [21] found that only 19% of participants performed better the 50-yards race after the short warm-up (2 × 45.7 m) when compared to the 44% who improved after the regular warm-up (~ 1200 m). On the other hand, Adams and Psycharakis [5] obtained no performance differences in the 100-m freestyle between 20- and 10-min warm-ups at 60% of maximum HR, thus suggesting that 10 min of activity was sufficient to prime the physiological mechanisms of the warm-up [1], and the selected intensity was not high enough to cause fatigue after the longer warm up [70]. Similarly, Neiva et al. [71] who compared a standard 1,200-m warm-up with shorter (600 m) and longer (1800 m) warm-ups did not find differences between the shorter and the standard warm-ups, while the 100-m time trial was 1.46% and 1.34% faster, respectively, when compared to the longer warm-up. Swimmers reached the lowest blood lactate concentration ([La^−^]) after the long warm-up, possibly because they achieved a better acid–base balance by stimulating buffering capacity with the prolonged low intensity activity [72]. In this sense, while the short and standard warm-ups involved durations of 15–20 min, the longer warm-up condition reached 30 min. Therefore, this would confirm that a certain duration of warm-up is required in swimming; however, long warm-ups may increase reliance on aerobic systems, which could be counterproductive for sprint swimming events, where anaerobic metabolism is a substantial energy source [71].

**Table 2:** In-water warm-ups of different volume (*n* = 5), or same volume and different intensity (*n* = 1)*

Reference	Participants, Sex & Age	Level & Experience	Control condition	Rest	Experimental condition	Rest	Main findings & results
Adams & Psycharakis [5]	8 males (20.1 ± 1.8 y)	Competitive	i) 20 min in-water warm-up including a freestyle base set, speciality stroke (containing kick, pull and drills), start and turns, before finishing with a 200 m swim down	20 min	ii) Mixed warm-up: 10 min in-water warm-up and 10 min sauna	20 min	The effects of the WU types (active or mixed) on 100-m at individual’s preferred stroke performance were not different. Possibly because of the long duration of the recovery period T100m: i: 61.1 ± 6.4 s; ii: 61.4 ± 6.7 s; The HR and RPE were lower after the active warm-up compared with the others
Balilionis et al. [21]	8 males (19.9 ± 0.6 y) 8 females (19.8 ± 0.7 y)	National Collegiate; > 5 years of competitive experience	i) ii) Short WU: 50-yards freestyle swim at 40% of their maximal effort and another 50 yards swim at 90% of their maximal effort (total: 100 yards)	3 min	ii) Regular WU; their own precompetitive swim WU (males: 1,257 ± 160 m) (females: 1,314 ± 109 m)	3 min	The best group-mean 50-yards freestyle mean times were performed after regular WUs. HR was higher before the 50-yards trial after the regular WU; T50-yard: i: 25.26 ± 1.61 s ii: 24.95 ± 1.53 s. HR: i: 142 ± 16 bpm; ii: 156 ± 23 bpm). RPE: i: 10.1 ± 1.7; ii: 12.1 ± 1.0. There were no differences for the swimming start variables
Houmard et al. [22]	8 males	Highly trained collegiate	i) 4 × 50-yards (110% $\dot{\text{V}}{\mathrm{O}}_{2\mathrm{max}}$)	5 min	ii) 1,500-yards at 65% $\dot{\text{V}}{\mathrm{O}}_{2\mathrm{max}}$; iii) 1,300-yards at 65% $\dot{\text{V}}{\mathrm{O}}_{2\mathrm{max}}$ + 4 × 50-yards at 110%$\dot{\text{V}}{\mathrm{O}}_{2\mathrm{max}}$	5 min	Although swimming time performance in 200-yards was not tested, they obtained higher distance per stroke (3.76%) in ii and iii. Higher lactate levels and HR were obtained after i
Mitchell & Huston [64]	10 males (19.3 ± 0.8 y)	Collegiate	i) 366-m swim at 70%$\dot{\text{V}}{\mathrm{O}}_{2\mathrm{max}}$	5 min	ii) 4 × 46-m swims at 1 min intervals at 110% $\dot{\text{V}}{\mathrm{O}}_{2\mathrm{max}}$	5 min	Performance in the tethered swim were slightly better in i; lactate was higher in ii Tethered swim: i: 137 ± 53.3 s; iii: 122.94 ± 37.2 s; lactate: i: 2.27 ± 0.81 mM/L; iii: 6.97 + 1.97 mM/L
Neiva et al. [71]	11 males (18.1 ± 3.3 y)	National-level (509 ± 63 FINA points); > 6 years of competitive experience	i) standard in-water WU of 1,200 m	10 min	ii) short warm-up 600 m iii) long warm-up 1,800 m	10 min	Swimmers were faster in 100-m freestyle after the short and moderate WU, suggesting that a long WU can impair the sprinting performance; specifically, the moderate WU showed higher swimming efficiency and an optimized recovery after the trial T100m: i: 59.29 ± 1.95 s; ii: 59.38 ± 2.18 s; iii: 60.18 ± 2.46 s; swimmers were 1.46—1.54% and 1.34—1.24% faster after i and ii, respectively, compared to iii
Neiva et al. [10] *	13 males (17.1 ± 1.5 y)	Competitive (567 ± 66 FINA points) 56.79 ± 2.24 s best T100m; 8.2 ± 1.5 years of training	i) 1,200 m: 300-m swim (100 m usual breathing, 100 m breathing in the fifth stroke, 100 m usual breathing) 4 × 100-m on 1:50 (2 × [25 m kick + 25 m increased stroke length]) + 8 × 50-m on 1:00 (2 × 50-m drill; 2 × 50-m building up velocity; 4 × [25-m race-pace set]; 100-m easy swim)	10 min	ii) 1,200 m: 300-m swim (100-m usual breathing, 100-m breathing in the fifth stroke, 100-m usual breathing) 4 × 100 m on 1:50 (2 × [25-m kick + 25-m increased stroke length]) + 8 × 50 m at 98–102% of critical velocity	10 min	No differences between WUs in 100-m front crawl. There were different biomechanical, physiological and psychophysiological strategies during the race on response to each condition T100m: i: 57.87 ± 1.84; ii: 57.83 ± 1.77 s (*d* = 0.07); T1st 50 m: i: 27.67 ± 0.99 s; ii: 27.70 ± 0.95 s (*d* = 0.7); T2nd 50 m: i: 30.31 ± 1.05 s; ii: 30.13 ± 0.92 s (*d* = 0.6); T15m: i: 6.74 ± 0.28 s; ii: 6.76 ± 0.29 s (*d* = 0.09); $\dot{\text{V}}{\mathrm{O}}_{2}$: i: 50.11 ± 5.79; ii: 50.95 ± 7.41 (*d* = 0.15); HR: i: 160 ± 15 bpm; ii: 163 ± 12 bpm (*d* = 0.5); lactate: i: 12.60 ± 2.50 mM/L; ii: 11.58 ± 3.11 mM/L (*d* = 0.56); core temperature: i: 37.50 ± 0.32 ºC; ii: 37.71 ± 0.35 ºC (*d* = 0.42); RPE: i: 18. ± 1.29; ii: 18.54 ± 1.20 (*d* = 0.82)

*y* Years old, *WU* Warm-up, T50–100 m Time performed in 50–100 m swimming, *d* = Cohen’s d (effect size), *SR* Stroke rate, *SL* Stroke length, *RPE* Rate of perceived exertion effort, *SWU* Standard warm-up

^*^Studies including different warm-ups with the same volume but different intensity

##### in-water Warm-Up Differing in Intensity

Prolonged high-intensity warm-ups may have negative effects on subsequent performances by reducing muscle glycogen levels and efficiency of fast-twitch muscle fibers [73]. This exponential decline is likely related to the expenditure of high-energy phosphate stores in active muscle, which reaches a plateau after 3–6 min [74]. Therefore, rest periods of at least ∼3–5 min after exercise are needed to allow a full PCr resynthesis [60, 61]. Only three studies have compared different warm-up intensities in swimming (Table 2). Houmard et al. [69] analyzed the effects of three warm-up intensities (i: 4 × 50-yards [110% $\dot{\text{V}}{\mathrm{O}}_{2\mathrm{max}}$]; ii: 1500-yards [65% $\dot{\text{V}}{\mathrm{O}}_{2\mathrm{max}}$]; iii: a combination of i and ii) over a distance of 400-yards, and concluded that the volume of the warm-up was much more relevant to prompt performance improvements than intensity. In this regard, it is not easy to draw conclusions between warm-ups that differ in intensity if they also differ in volume or duration, as these loading parameters also are relevant for eliciting the cardiovascular effects of the warm-up [1, 2]. Thus, studies testing different warm-up intensities should equate volume and ensure sufficient durations (no lesser than 5–10 min) to favor adequate cardiovascular responses and comparisons. For instance, Mitchell and Huston [64] found no difference in the 200-yards time between the 200-yards warm-up performed at 110% of $\dot{\text{V}}{\mathrm{O}}_{2\mathrm{max}}$ and the 400-yards warm-up performed at 70% of $\dot{\text{V}}{\mathrm{O}}_{2\mathrm{max}}$. In this case, the high-intensity warm-up was probably too short and thus insufficient to increase core and muscle temperatures [1].

Neiva et al. [10], compared two warm-ups of similar volume (1200 m), but differing in intensities only in the last bout: i) 4 × 50 m at 100-m freestyle race-pace intensity; ii) 4 × 50 m at 98–102% of critical speed (i.e., aerobic predominance). The results showed trivial differences (*d* = 0.07) between the race-pace and aerobic warm-ups in the 100-m performance. However, swimmers were able to reach a higher stroke rate and [La-] values after the race-pace warm-up, and higher values of stroke length and propulsive efficiency after the aerobic warm-up, which was in agreement with the previous findings by Houmard et al. [69]. Possibly, as the increased excitability of motor neurons due to higher speed may improve the rate of force development and power production [75], this may have increased the stroke rate after the race-pace warm-up as the swimming speed was almost maximal. In this regard, Neiva et al. [68] suggested that warm-up exercises could influence biomechanics in swimming as occurs in other sports. Thus, a different physiological or biomechanical pattern may be primed depending on the warm-up intensity. In any case, it is possible that, given the high volume of low intensity training undertaken by swimmers on a daily basis [76], they may develop a high proportion of slow-twitch fibers that may not respond to high intensity stimuli as other athletes with a higher fast-twitch fiber percentage [77, 78]. Hence, this could be a possible reason in favor of including low intensity and moderate to high mileage during warm-ups to trigger positive acute performance adaptations in swimmers.

###### Summary

Despite the great variability in athletes' responses to warm-up, it appears that a warm-up involving moderate activity (1000 m at 60–80% $\dot{\text{V}}{\mathrm{O}}_{2\mathrm{max}}$) followed by a reasonable rest period (3–10 min) is more beneficial to maximize short-term swimming performances. In this regard, a longer or higher intensity warm-up may lead to undesired activation of inappropriate energy systems, while potentially mediating different biomechanical and physiological responses that may be positive or negative depending on the demands of the next task.

#### In-Water Warm-up Including External Load Elements

Only two recent studies have compared the effects of a 1000–1200 m in-water warm-up with the same warm-up followed by several swimming bouts using external elements to verify PAPE responses (see Table 3). Thus, Barbosa et al. [57], evaluated the effects of 8 × 12.5 m maximal swimming efforts with hand-paddles and parachute and reported reductions in maximal force and impulse after two and six min of rest. These authors [57] concluded that the CA was detrimental and that fatigue and the reduced rest interval masked the possibly effects of PAPE. More recently, Abbes et al. [79], evaluated the effects of 3 × 10 s fully tethered efforts performed with hand-paddles and observed deteriorations in 50-m performance time and stroke lengths after 8 min of rest. These authors [79] suggested that the lack of results resulted from an inadequate rest time provided to the sample of regional-level adolescent swimmers. However, another possible reason may be related to kinematic changes and body positioning expected during stationary tethered swimming that could lead to a different trajectory of the hands when compared to actual swimming [80, 81]. These changes, combined with those induced by the hand paddles [82], could profoundly affect how the swimmer feels the water and therefore not properly replicate the biomechanical requirements of swimming, affecting subsequent performance.

**Table 3:** In-water warm-up including external load elements (*n* = 5)

Reference	Participants, Sex & Age (mean ± SD)	Level & Experience	Control condition	Rest	Experimental condition	Rest	Main findings & results
Abbes et al. [79]	14 males (13.0 ± 2.0 y)	Regional, 520 ± 98 FINA points; 4 years (6 h/week), familiar with push-ups, squat jumps and burpees	i) 1,200-m in-water WU + 200 m freestyle at moderate pace	30 min	ii) i + 3 × 10-s tethered swimming using paddles (1 min rest in-between)	20 min + PAP + 10 min	The experimental protocol evoked a deterioration in 50-m front crawl performance in young swimmers T50m: i) 32.48 ± 3.35 s; ii) 32,68 ± 3,68 s (*d* = 0.3); SL: i) 1,58 ± 0,53 m; ii) 1,57 ± 0,52 m; RPE: i) 8 ± 1; ii) 7 ± 1; [La-] PostWU: i) 1,78 ± 0,86 mM/L; ii) 2,92 ± 0,97; [La-]_1min: i) 6,8 ± 1,76 mM/L; ii) 6,55 ± 1,89; [La-]_3min: i) 6,91 ± 1,81 mM/L; ii) 6,75 ± 2,38 mM/L
Barbosa et al. [57]	5 males & 3 females (18.4 ± 1.3 y)	Well-trained national competitive; experienced with in-water strength training	i) 1,000-m in-water WU	2.5 and 6.5 min (4 min after)	ii) 8 × 12.5-m maximal swimming efforts using hand paddles and parachute	2.5 and 6.5 min (4 min after)	The CA was detrimental. The weaker swimmers deteriorated performance more than the stronger ones Peak force i: ~ 215 N; ii_2.5 min: ~ 200 N; ii_6.5 min: ~ 205 N (*n*^2^ = 0.53); impulse: i: 76 N·s; ii_2.5 min: ~ 70 N·s; ii_6.5 min: ~ 70 N·s (*n*^2^ = 0.72); The impulse level correlated with the pre-post changes in peak force (*r* = 0.76) and RFD (r = 0.76)
Cuenca-Fernández et al. [34]	20 males (18.0 ± 1.4 y)	Competitive; T50m 74.29 ± 7.89% WR (477 ± 163 FINA points) 1 national participation in the last year	i) 400 m in-water WU (2 × 100 m easy with 2 starts; 1 × 50 m front crawl swim (12,5 fast/12.5 smooth); 1 × 50 race-pace; 100 m easy) + 2 × 10 reps dynamic stretching protocol (forward leg swings, ankle-dorsi and plantar-flexion, side leg signs, high knees, heel flicks, squats and lunges)	6 min	ii) incremental semi-tethered resisted swimming test (10, 20 30 and 40% of the maximal power load)	6 min	Swimmers benefited from semi-tethered resisted swimming to develop high power and propulsive impulse in a 20 m freestyle effort, due to adaptive neuromuscular changes Force: i: 42.95 ± 10.15 N; ii: 43.22 ± 10.13 N; impulse: i: 4.41 ± 1.54 N·s; ii: 4.48 ± 1.58 N·s; power: i: 49.98 ± 15.40 W; ii: 51.38 ± 14.93 W; RFD: i: 31.29 ± 13.70 N/s; ii: 31.79 ± 13.49 N/s; velocity: i: 1.17 ± 0.12 m/s; 1.21 ± 0.14 m/s; SR: i: 61.56 ± 7.07cyc/min; ii: 61.43 ± 7.27cyc/min; SL: i: 1.21 ± 0.15 m; ii: 1.23 ± 0.16 m; Distance in 5 strokes: i: 5.77 ± 0.72 m; ii: 1.23 ± 0.16 m; T5m: i: 4.23 ± 0.57 s; ii: 4.19 ± 0.56 s
Hancock et al. [59]	15 males (20.1 ± 1.0 y) 15 females (20.0 ± 0.9 y)	Varsity team; 7 had a sprint training background; 7 had a distance training background and 16 had a mix of sprint and distance training background	i) 900 m in-water WU (800 m freestyle swim proceeded by 4 × 25 sprints [40 s of work + recovery])	6 min	ii) i + 4 semi tethered resisted swimming sprints attached to a Power Rack (The individualized load was calculated and corrected by the body mass and the 100-m best time × 0.2 to bring the effort within a 7-s of duration [derived from the power rack])	6 min	Semi-tethered resisted swimming enhanced 100 m freestyle performance. There were no sex-regulated PAP responses Males: T100m: i: 59.47 ± 2.56 s; ii: 59.05 ± 2.55 s; T50m: i: 27.89 ± 1.07 s; ii: 27.67 ± 1.18 s; T50-100 m: i: 31.59 ± 1.56; ii: 31.38 ± 1.52 s; Females: T100m: i: 67.42 ± 4.39 s; ii: 66.78 ± 3.80 s; T50m: i: 31.67 ± 1.98 s; ii: 31.36 ± 1.61 s; T50-100 m: i: 35.75 ± 2.46; ii: 35.42 ± 2.24 s; La^−^: i: 11.5 mMol/L; ii: 12.3 mMol/L
Juarez et al. [83]	18 males (16.2 ± 3.8 y)	Competitive; 6 years of experience in swimming and 2 years in resistance training	i) 1,000 m in-water WU at low intensity, 100 m at higher intensity, and 100 m at low intensity	30 s	ii) 4 × (semi-tethered resisted sprint (12 m)—30% of maximal power load)	4 × 30 s	Semi-tethered resisted swimming did not improve performance in 25 m freestyle swimming. Analyzing the individual responses, high variability was observed, with participants decreasing/increasing their swimming times Pre: i: 14.85 ± 1.72 s; ii: 14.55 ± 1.54 s; Post_1: i: 14.72 ± 1.61 s. ii: 14.44 ± 1.49 s (∆ = 0.75%); Post_2: i: 14.80 ± 1.68; ii: 14.49 ± 1.66 s (∆ = 0.41%); Post_3: i: 14.84 ± 1.72 s; ii: 14.35 ± 1.49 s (∆ = 1.37%); Post_4: i: 14.81 ± 1.60; 14.35 ± 1.52 s (∆ = 1.37%)

*y* Years old, *WU* Warm-up, *WR* World Record, T25-50–100 m: Time performed in 25–50–100 m swimming, *CA* Conditioning activity; *d* = Cohen’s d (effect size), [La-]: Blood lactate concentration, *SR*: Stroke rate, *SL*: Stroke length, *ML* Maximal Load, *RFD* Rate of force development, *PAP* Post-activation potentiation

On the other hand, three studies have tested in-water conditioning methods resulting from the swimmers’ effort to overcome a resistance while swimming (Table 3). Previously, Juarez et al. [83], compared the differences of an in-water warm-up of 1200 m (10% at high-intensity), with the same warm-up followed by 4 sets of semi-tethered resisted swimming over 12 m (30% of the individual maximum load). There were no significant changes in performance in 25 m assessed 30 s after each semi-tethered trial (i.e., 4 × 25 m), with only some trends (*p* > 0.1; ∆ = − 0.9%) to improvement after the resisted efforts. As some participants decreased and others increased their performance times, the authors [83] argued that significant enhancements could perhaps be achieved for the whole group with longer rest times. Subsequently, Hancock et al. [59] compared a 900 m in-water warm-up including 4 × 25 m sprints with the same warm-up including 4 × 25 m moderate-intensity resisted sprints attached to a pulleys system. After 8 min of rest, 100-m freestyle performances were significantly improved after the resisted sprint condition and these authors [59] suggested that pulling low loads may induce an increase in muscle stiffness, thus favoring temporary neurological and mechanical adaptations [84]. More recently, Cuenca-Fernández et al. [34] compared a 400-m in-water warm-up with an incremental semi-tethered resisted protocol of 15 to 20 s-bouts at 10, 20, 30 and 40% of the individual maximum power load. These authors [34] observed that after 6 min of rest, there were increases in speed, force, and impulse, and in other kinematic variables such as stroke-length (*r* = 0.66), and time to 5 m (*r* = − 0.72). Hence, considering these 3 studies in conjunction, it can be suggested that submaximal efforts as semi-tethered resisted swimming could be a valid means to improve performance of swimmers as a consequence of PAPE responses which are the result of an optimized balance between fatigue and potentiation [31].

##### Summary

Compared to a standard in-water warm-up (~1000m), tethered swimming would apparently not be considered as an appropriate stimulus to generate effective PAPE responses. Further, the use of hand-paddles during warm-ups does not appear to provide superior muscular responses to those achieved by traditional in-water warm-ups. The fact that neither of these methods elicits positive responses may possibly be due to changes in stroke pattern caused by the tethered swim and a loss of feeling for the water with the use of the paddles. In contrast, the three found studies which focused on semi-tethered resisted methods have obtained positive outcomes, possibly because of PAPE responses, and without altering the swimming technique.

#### In-Water Combined with Dryland Warm-up Exercises

Many researchers and coaches have experimented with dryland methods in swimming to try to enhance the improvements obtained in the neuromuscular system after the in-water warm-up (Table 4). Over the next subsections, we will summarize all the different proposals found in the literature.

**Table 4:** In-water warm-up combined with dryland conditioning exercises during the transition phase (*n* = 19)

Reference	Participants, Sex & Age	Level & Experience	Control condition	Rest	Experimental condition	Rest	Main findings & results
Abbes et al. [56]	17 males (13.0 ± 2.0 y)	Regional, 520 ± 98 FINA points; 4 years (6 h/week), familiar with push-ups, squat jumps and burpees	i) 1,200-m in-water WU	30 min	ii) i + 30-s maximal push-ups; iii) i + 30-s squat jumps; iv) i + 30-s burpees;	20 min + PAP + 10 min	None of the experimental protocols showed improvements in 50-m freestyle performance T50m: i) 32.84 ± 2.53 s; ii) 32.62 ± 2.81 s, *d* = 0.08; iii) 32.42 ± 2.32 s, *d* = 0.17; iv) 32.46 ± 2.26 s (*d* = 0.15); SR: i) 0.79 ± 0.07; ii) 0.8 ± 0.08; iii) 0.81 ± 0.08; iv) 0.8 ± 0.08; SL: i) 1.96 ± 0.20; ii) 1.93 ± 0.25; iii) 1.92 ± 0.24; iv) 1.94 ± 0.22; RPE: i) 8.0 ± 1.0; ii) 8.3 ± 1.5; iii) 8.5 ± 1.0; iv) 8.0 ± 0.2; La^−^ was higher after the 50 m race on the PAP groups (*d* = 0.68)
Barbosa et al. [97]	12 males (23.5 ± 3.3 y)	Skilful competitive; 8.08 ± 4.59 h/week	i) 1,400-m in-water WU: 400-m in self-selected stroke and pace, 200-m of front-crawl drills (25-m steady/25-m fast), 200-m of flutter kick using a kickboard (15-m fast/35-m steady), 4 × 100 m (2 front-crawls and 2 individual medleys with 10-s rest in between), 100-m (easy) and 2 × 50-m (dive followed by 15-m fast/35-m easy) of front-crawl drills	8 min	ii) 700-m in-water warm-up + 2 × 5 arm-pulls with resistance bands. Resistance band level was chosen on individual basis during the familiarization session (light, medium, or heavy; resistance range: 3.17–19.50 kg, 4.53–22.68 kg and 7.27–26.76 kg, respectively)	5 min + PAP + 8 min	The CA elicited a large improvement on arm-thrust, but with small improvement in the 25-m freestyle performance Increases in peak thrust: i: 72.3 ± 11.6; ii: 80.9 ± 11.9 (∆ = 13.37%. *d* = 0.50); increases in thrust-time integral: i: 33.5 ± 8.6; ii: 38.3 ± 6.2 (∆ = 18.73%. *d* = 0.74); increases in speed: i: 0.84 ± 0.10; ii: 0.86 ± 0.09 (∆ = 2.78%. *d* = 0.18); increases in speed fluctuation: i: 0.14 ± 0.02; ii: 0.14 ± 0.05 (∆ = 0.73%. *d* = 0.04)
Crespo et al. [90]	10 males (16.6 ± 2.0 y) 7 females (15.4 ± 1.8 y)	Competitive; males: 29.64 ± 2.46 s FINA: 402 ± 120; females: 31.36 ± 1.93 s FINA: 483 ± 102 (50 m long course); > 5 years of experience	i) Dynamic stretching followed by 600-m in-water WU (400-m easy swim; 4 × 25-m strong; 50 ventral kick built; 3 × 10-m UUS built)	5 min	ii) i + 4 maximum half squat reps on an inertial flywheel	5 min	The WU which included CA through a flywheel device, showed better results in UUS performance. Males obtained improvements in more variables than females Time to 10 m; males i) 5.77 ± 0.44 s; ii) 5.64 ± 0.46 s (*d* = 1.03); females: i) 6.34 ± 0.80 s; ii) 6.09 ± 0.66 s (*d* = 1.24); Push-off velocity: males: i) 2.60 ± 0.21 m/s; ii) 2.68 ± 0.16 m/s (*d* = 1.20)
Cuenca-Fernández et al. [85]	10 males & 4 females (17 – 23 y)	National competitive; 5 years of participation in national competition	i) 400-m in-water WU (2 × 100-m easy with 2 starts; 1 × 50-m front crawl swim [12.5 fast/12.5 smooth]; 1 × 50-m race-pace; 100-m easy) + 2 × 10 reps dynamic stretching protocol (forward leg swings, ankle-dorsi and plantar-flexion, side leg signs, high knees, heel flicks, squats and lunges)	8 min	ii) i + 3 lunge reps—85% ML; iii) i + 3 maximal eccentric flywheel reps	8 min	The WU which included CAs through maximal loaded lunges, or through a flywheel device, showed better results in a swimming start performance DD: i) 294.20 ± 8.67 cm; ii) 300.29 ± 8.65 cm; iii) 304.28 ± 9.06 cm; FT: i) 0.33 ± 0.14 s; ii) 0.31 ± 0.15 s; iii)0.28 ± 0.13 s; VxH: i) 3.63 ± 0.11 m/s; ii) 4.15 ± 0.12 m/s; iii) 4.89 ± 0.12 m/s; T5m: i) 1.75 ± 0.05 s; ii)1.71 ± 0.05 s; iii) 1.65 ± 0.04 s; T15m: i) 7.54 ± 0.23 s; ii) 7.40 ± 0.21 s; iii) 7.36 ± 0.22 s; BT: 0.79 ± 0.01 s; ii) 0.78 ± 0.03 s; iii) 0.74 ± 0.02 s
Cuenca-Fernández et al. [86]	11 males (18.9 ± 0.7 y) 2 females (19.0 ± 0.7 y)	Competitive; 5 years of national level competitive participation	i) 400-m in-water WU (2 × 100-m easy with 2 starts; 1 × 50 m front crawl swim (12,5 fast/12.5 smooth); 1 × 50-m race-pace; 100-m easy) + 2 × 10 reps dynamic stretching protocol (forward leg swings, ankle-dorsi and plantar-flexion, side leg signs, high knees, heel flicks, squats and lunges)	6 min	ii) i + 4 maximal eccentric flywheel reps	6 min	The WU which included CA through a flywheel device, produced higher vertical ground reaction force values which resulted in better results in swim start performance Average vertical force: i: 27.18 ± 144.14 N; ii: 58.28 ± 195.27 N (*d* = 0.18); peak vertical force: i: 509.55 ± 105.26 N; ii: 551.79 ± 106.43 N (*d* = 0.39); resultant impulse: i: 251.27 ± 34.41 N·s; ii: 267.09 ± 38.17 N (*d* = 0.43); resultant velocity: i: 3.93 ± 0.60 m/s; ii: 4.32 ± 0.88 m/s (*d* = 0.51); RFD: i: 3261.16 ± 2029.73 N/s; ii: 3780.39 ± 2675.87 N/s (*d* = 0.21)
Cuenca-Fernández et al. [53]	17 males (18.4 ± 1.4 y)	Regional and national-level (T50m—74.26% of WR); 5 years of participation in regional-national competition	i) 400-m in-water WU (2 × 100-m easy with 2 starts from the wall; 1 × 50-m front crawl swim (12,5 fast/12.5 smooth); 1 × 50-m race-pace; 100-m easy) + 2 × 10 reps of dynamic stretching protocol (forward leg/arm swings, ankle-dorsi and plantar-flexion, arm circles, side leg swings, arm crossovers, high knees, hand up, heel flicks, squats and lunges)	6 min	ii) i + 3 maximal eccentric flywheel reps and 3 arm-pull; iii) i + 3 lunge and 3 arm-pull reps—85% RM	6 min	The WU which included maximal load or eccentric CAs, showed better results in a swimming start performance. However, these CAs were inappropriate or produced fatigue on 50-m freestyle swimming DT: i: 0.93 ± 0.09 s; ii: 0.93 ± 0.10 s; iii: 0.94 ± 0.13 s; DD: i: 3.11 ± 0.26 m; ii: 3.20 ± 0.32 m; iii: 3.14 ± 0.29 m; DV: i: 3.26 ± 0.33 m/s; ii: 3.40 ± 0.49 m/s; iii: 3.31 ± 0.47 m/s; T5m: i: 1.57 ± 0.11 s; ii: 1.52 ± 0.13 s; iii: 1.52 ± 0.13 s; T15m: i: 7.19 ± 0.54 s; ii: 7.05 ± 0.66 s; iii: 7.04 ± 0.57 s; T50m: i: 27.28 ± 1.42 s; ii: 27.51 ± 1.43 s; iii: 27.31 ± 1.45 s; SR: i: 57.02 ± 6.93 cyc/min; ii: 55.30 ± 6.21 cyc/min; iii: 55.99 ± 6.43 cyc/min; SL: i: 1.76 ± 0.21 m; ii: 1.69 ± 0.25 m; iii: 1.72 ± 0.25 m
Cuenca-Fernández et al. [34]	20 males (18.0 ± 1.4 y)	Competitive; T50m 74.29 ± 7.89% WR (477 ± 163 FINA points) 1 national participation in the last year	i) 400 m in-water WU (2 × 100 m easy with 2 starts from the wall; 1 × 50 m front crawl swim (12,5 fast/12.5 smooth); 1 × 50-m race-pace; 100-m easy) + 2 × 10 reps dynamic stretching protocol (forward leg swings, ankle-dorsi and plantar-flexion, side leg signs, high knees, heel flicks, squats and lunges)	6 min	ii) i + 3 arm-pull reps—85% RM; iii) an incremental semi-tethered resisted swimming test (10, 20 30 and 40% of the maximal power load)	6 min	PAPE responses were obtained after high-resistance pull-over repetitions in 20-m semi-tethered swimming; however, swimming performance was not improved, possibly due to stroke alterations Force: i: 42.95 ± 10.15 N; ii: 41.82 ± 9.87 N; iii: 43.22 ± 10.13 N; impulse: i: 4.41 ± 1.54 N·s; ii: 3.49 ± 1.39 N·s; iii: 4.48 ± 1.58 N·s; power: i: 49.98 ± 15.40 W; ii: 42.48 ± 12.95 W; iii: 51.38 ± 14.93 W; RFD: i: 31.29 ± 13.70 N/s; ii: 34.52 ± 16.55 N/s; iii: 31.79 ± 13.49 N/s; velocity: i: 1.17 ± 0.12 m/s; ii: 1.01 ± 0.15 m/s; iii: 1.21 ± 0.14 m/s; SR: i: 61.56 ± 7.07 cyc/min; ii: 64.70 ± 9.84 cyc/min; iii: 61.43 ± 7.27 cyc/min; SL: i: 1.21 ± 0.15 m; ii: 0.97 ± 0.20 m; iii: 1.23 ± 0.16 m; Distance covered in 5 strokes: i: 5.77 ± 0.72 m; ii: 0.97 ± 0.20 m; iii: 1.23 ± 0.16 m; T5m: i: 4.23 ± 0.57 s; ii: 5.22 ± 0.88 s; iii: 4.19 ± 0.56 s
Dalamitros et al. [45]	10 males (19.3 ± 2.2 y) 9 females (18.1 ± 1.9 y)	National-level (top 8) ~ 560 FINA points (2016) 50 m front crawl; 9 to 12 years of competitive experience	i) 1,000-m in-water WU: 300 m swim (smooth); 6 × 50-m swim (1:15, pull, kick, drill); 8 × 25-m (1:00, 4 × 25 m: 12.5-m at 90% race pace followed by 12.5-m easy and 4 × 25-m vice versa); 2 × 50-m (2:00, 25-m all-out followed by 25-m easy pace); 100-m easy swim	30 min seated	ii) i + 2 × (3 × med ball throw downs (2 kg), 3 × med ball side to side crunches (2 kg) and 3 × 40 cm box jumps); iii) i + 7 dynamic stretching exercises with a 10:10 s work-to-rest ratio (3 for upper body: arm circles, lateral arm swings, and bend over opposite arm swings), 2 for the body core (twisting toe touch and arm downside bending), and 2 for the lower body (frontal plane leg swings and with a 90-degree knee angle)	15 min + CAs + 10 min	The WU which included CAs obtained better results on 50-m freestyle. However, different PAPE responses were obtained depending on the sex Males: T50m: i: 27.34 ± 0.91 s; ii: 26.89 ± 1.09 s (d = 0.29); iii: 27.25 ± 1.35 s; SL. SI. HR and RPE were no different. although observable higher values were obtained in SR in ii Females: T50m: i: 31.15 ± 1.00 s; ii: 31.46 ± 1.03 s; iii: 30.93 ± 1.11 s (d = 0.31); SR, SI, HR and RPE were no different, although observable higher values were obtained in SL in iii
Dalamitros et al. [96]	22 males Trained (20.3 ± 1.8 y) Untrained (21.8 ± 0.8 y)	Trained: Within the national top 8 (Training: 18.0 ± 2 h/week); Untrained: Nonactive athletes with a wide variety of swim training background	i) 1,100-m in-water WU (continuous swimming/arm and kick drills/short sprints/cool down)	20 min seated	ii) 600-m in-water warm-up (continuous swimming/arm and kick drills/short sprints/cool down) + 15 min rest + 5 loaded box jumps (weighted best—10% of BW)	4, 8 or 12 min (individually applied)	The 50-m breaststroke variables tested after the CAs were not influenced by the different competitive level of the participants Trained group: T25m: i: 17.1 ± 1.4 s; ii: 6.8 ± 1.4 s (d = 0.24); T50m: i: 29.0 ± 3.1 s; ii: 28.6 ± 3.8 s (d = 0.13); stroke count: i: 29.0 ± 3.1; ii: 28.6 ± 3.8 (d = 0.13); RPE: i: 6–7; ii: 6–7; saturation O2: i: 69.5 ± 13.5; ii: 73.2 ± 11.2 (*d* = 0.28); HR: i: 156.6 ± 13.5 bpm; ii: 157.7 ± 13.2 bpm (*d* = 0.05) Untrained group: T25m: i: 19.3 ± 2.6 s; ii: 18.5 ± 2.3 s (*d* = 0.21); T50m: i: 41.9 ± 5.5 s; ii: 41.5 ± 5.3 s (*d* = 0.06); stroke count: i: 32.7 ± 6.2; ii: 33.1 ± 6.6 (*d* = 0.06); rpe: i: 6–7; ii: 6–7; saturation O2: i: 71.9 ± 18.4; ii: 69.4 ± 14.4 (*d* = 0.16); HR: i: 161.5 ± 9.1 bpm; ii: 163.6 ± 9.0 bpm (*d* = 0.22)
de Arruda et al. [88]	13 males (19.4 ± 3.4 y)	Competitive; 3 years of experience (5 h/week) (50 m time—77% WR). Familiarized with the CE	i) Standardized in-water WU (30 min)	10 min	ii) i (15 min) + lunges (3 × 85% RM); iii) i (15 min) + pull-ups (3 max reps) and box jumps (1 × 5 with 10% BW); iv) ii + iii	4, 8 or 12 min (individually applied)	The CAs did not improve 50-m front crawl compared to the standard WU. Nevertheless, the CEs appeared to influence each phase of the event differently DD: i: 375.95 ± 25.91 cm; ii: 383.56 ± 24.73 cm (d = 0.30); iii: 380.80 ± 28.46 cm (*d* = 0.18); iv: 382.38 ± 30.29 cm (d = 0.23); VxH: i: 4.39 ± 0.84 m/s; ii: 3.22 ± 1.70 m/s (*d* = -0.92); iii: 4.05 ± 0.80 m/s (*d* = -0.41); iv: 4.09 ± 0.95 m/s (*d* = -0.33); T5m: i: 1.57 ± 0.40 s; ii: 1.39 ± 0.19 s (*d* = -0.59); iii: 1.46 ± 0.16 s (d = -0.40); iv: 1.37 ± 0.10 s (*d* = -0.77); T15m: i: 7.59 ± 0.35 s; ii: 7.58 ± 0.50 s (d = -0.02); iii: 7.69 ± 0.47 s (d = 0.24); iv: 7.53 ± 0.47 s (*d* = -0.14); T50m: i: 27.01 ± 1.25 s; ii: 27.17 ± 1.18 s (*d* = 0.20); iii: 27.44 ± 1.26 s (*d* = 0.41); iv: 27.12 ± 1.44 s (*d* = 0.17); SR: i: 1.43 ± 0.15 Hz; ii: 1.75 ± 0.22 Hz; iii: 1.76 ± 0.26 Hz; iv: 1.74 ± 0.19 Hz; SL: i: 1.04 ± 0.17 m; ii: 1.06 ± 0.15 m; iii: 1.11 ± 0.19 m; iv: 1.06 ± 0.11 m
Ðurovic et al. [87]	10 males (16 ± 2 y)	National; 5 years of experience (8 h/week) Dryland conditioning (1 h/week)	i) 1,600 m in-water WU: 400 m free/back light swim, 2 × 100 m medley; 200 m flutter kicking, 4 × 50 m front-crawl (2 easy 2 medium), 4 × 50 m front-crawl (dive fast to 15 m and 35 m easy), and 200 m easy using fins	8 min	ii) 10 min of light skipping, dynamic stretching, and general movement + 3 × 5 DJ from a box (40 cm) iii) i + 3 × 5 DJ from a box (40 cm)	8 min	The DJ protocol, in addition to in-water WU, is an effective tool to improve athlete’s capacity at the swim start to 15 m (2.31%) the eRFD (12.83%) and the IES (12.43%). T15m: i: 7.47 ± 0.10 s; ii: 7.41 ± 0.12 s; iii: 7.31 ± 0.11 s; eRFD: i: 66.75 ± 4.61 N·s; ii: 72.31 ± 3.17 N·s; iii: 75.31 ± 5.39 N·s; IES: i: 41.81 ± 1.64; ii: 45.88 ± 1.92; iii: 47.01 ± 1.81
Juarez et al. [83]	18 males (16.2 ± 3.8 y)	Competitive; 6 years of experience in swimming and 2 years in resistance training	i) 1,000 m in-water WU at low intensity, 100 m at higher intensity, and 100 m at low intensity	30 s	ii) i + 4 series of elastic bands	4 × 30 s	The elastic bands protocol did not improve performance in 25-m freestyle swimming Pretest: i: 14.85 ± 1.72 s; ii: 14.71 ± 1.52 s; Post_1: i: 14.72 ± 1.61 s. ii: 14.85 ± 1.41 s; Post_2: i: 14.80 ± 1.68; ii: 14.81 ± 1.35 s; Post_3: i: 14.84 ± 1.72 s; ii: 14.68 ± 1.33 s; Post_4: i: 14.81 ± 1.60; 14.85 ± 1.20 s
McGowan et al. [47]	11 males & 5 females (16 ± 1 y)	National junior (100 m time: 59.41 ± 3.48 s)	i) 1,300 m in-water WU (400 m Freestyle easy); 3 × 100 m medley (100 m: kick, drill, swim); 3 × 100 m freestyle (80,90,95% race-pace); 4 × 50-m (15-m race pace, 35-m easy); 4 × 25-m freestyle (dive start, race pace)	30 min seated with the only activity to change into their racing suit	ii) i + wearing heating elements iii) i + 5 min dry-land-based exercise routine [2 × (3 × medicine ball throw downs (2 kg), 3 × 10 s simulated butterfly kicks and 3 × 0,4 m box jumps)] iv) iii + wearing heating elements	15 min	An improvement in 100-m freestyle performance was demonstrated when dryland-based activation exercises were completed alone (∼0.7%), and in combination with the wearing of a heated tracksuit jacket (∼1.1%) T100m: i: 60.7 ± 3.36 s; ii: 60.37 ± 3.15 s (*d* 0.12); iii: 60.26 ± 3.50 s (*d* = 0.18); iv: 59.9 ± 3.7 s (*d* = -0.27); T15m: i: 7.23 ± 0.17 s; ii: 7.03 ± 0.24 s (*d* = 0.45); iii) 7.13 ± 0.16 s (*d* = -0.05); iv) 6.86 ± 0.19 s (*d* = 0.92); skin temperature pre time trial: i: 33.1 ± 0.3 ºC; ii: 33.9 ± 0.3ºC; iii: 33.3 ± 0.3ºC; iv: 34.3 ± 0.1ºC. There were no differences in La^−^ and HR
Nepocatych et al. [23]	4 males (37 ± 10 y) 6 females (34 ± 8 y)	Master (Best 50-yd time: Males: 29,5 ± 7,0; Females: 26,3 ± 3,3); > 3 years of experience, ≥ 3practice/week	i) 500-yards in-water WU including at least 2 × 25-yards sprints at 90%	3 min	ii) 100-yards freestyle swim (50-yards at 40% and 50-yards at 90%) + 5 × 1 min upper body vibration (22 Hz) including arm pull on a swim bench iii) 5 × 1 min upper body vibration (22 Hz) including arm pull on a swim bench	3 min	Swimmers could perform better in 50-yards after acute upper body vibration combined with in-water swimming and arm-pull warm-up routine 50-yards time: i: 29.1 ± 3.36 s; ii: 28.9 ± 3.39 s; iii: 29.1 ± 3.55 s; RPE: i: 17 ± 2; ii: 16 ± 2; iii: 16 ± 1; HR: i: 148 ± 15 bpm; ii: 138 ± 14 bpm; iii: 139 ± 12 bpm; stroke-count: i: 35 ± 7; ii: 35 ± 5; iii: 36 ± 6
Ng et al. [89]	16 males (22.1 ± 3.8 y)	Competitive; 7.4 ± 4.1 years of competitive experience	i) 1,400-m in-water WU (400-m self-selected stroke and pace; 200 m front-crawl drills (25 m steady/25 m fast), 200 m flutter kick drills (15 m/35 m steady), 4 × 100-m (2 front-crawls and 2 medley with 10-s rest in between), 100-m (easy) and 2 × 50-m (dive followed by 15 m/35 m easy) of front crawl drills	8 min	ii) 700-m in-water warm-up (half of the exercises/distances performed in i) + 5 min of rest + 2 × 5 counter movement jump (CMJ) with body weight	8 min	There were improvements in 25-m flutter kick thrust, kinematics, and performance, when participants added CMJs after the in-water WU Speed: i: 0.59 ± 0.10 m/s; ii: 0.66 ± 0.13 m/s (11.60%. *d* = 0.54); kicking freq: i: 2.40 ± 0.24; ii: 2.48 ± 0.32 (3.17%. *d* = 0.27); peak thrust: i: 92.7 ± 15.8; ii: 105.2 ± 21.1 (15.14%; *d* = 0.66; mean thrust: i: 35.52 ± 7.42; ii: 39.56 ± 12.44 (14.60%. *d* = 0.40); thrust-time integral: i: 9.89 ± 1.71; ii: 9.63 ± 2.44 (0.13%. *d* = 0.12)
Ramos-Campo et al. [15]	7 males & 6 females (15.1 ± 2.1 y)	Competitive (T100m = 72.0 ± 11.8); > 8 years of training (6 h/week)	i) 1,000-m in-water WU (300 freestyle easy; 4 × 50 drills; 4 × 50 freestyle [15-m race-pace, 35-m easy]; 4 × 25-m freestyle [dive-start, race-pace] and 200-m freestyle easy)	30 min (rest in normoxia)	ii) i + 30 min rest in hypoxia; iii) i + 10 min rest + 5 min dryland-based circuit in normoxia; iv) i + 10 min rest + dryland circuit in hypoxia; 2 × (3 × med ball throw-downs [2 kg], 3 × 10 simulated underwater kick holding a BodyBlade oscillation device above the head, and 3 × horizontal jump)	5 min rest after the dryland circuit	A dryland-based exercise re-warm-up routine, under hypoxic conditions, attenuated the decline of tympanic temperature during a 30 min transitional phase, thus improving 100-m time trial performance in competitive swimmers T100m: i: 75.7 ± 6.7 s; iii: 75.2 ± 6.7 s; ii: 75.0 ± 6.4 s; iv: 73.4 ± 6.2 s; Saturation O2: i: 97.5 ± 1.0; iii: 97.8 ± 0.7; ii: 90.8 ± 4.6; iv: 87.5 ± 3,0; tympanic temperature: i: 35.9 ± 0.6; iii: 36.3 ± 0.4; ii: 36.0 ± 0.4; iv: 36.3 ± 0.4; HR and RPE did not present differences
Ruiz-Navarro et al. [91]	44 males (15.2 ± 1.4 y) 48 females (14.4 ± 1.5 y)	National; > 3 years of experience; training 12–15 h/week including dryland work	i) dynamic stretching protocol followed by 400 m of varied swimming	10 min	ii) i + 10 min rest + 4 Tuck Jumps	< 1 min	The experimental WU did not show any significant effect on UUS performance or kinematics. No specific responses were obtained from the PAPE when differentiating by sex and/or level of strength of the participants Push-off vel: Males i: 2.96 ± 0.33 m/s; ii: 3.00 ± 0.43 m/s; Females: i: 2.53 ± 0.29 m/s; ii: 2.55 ± 0.33 m/s UUS velocity: Males: i: 1.35 ± 0.19 m/s; ii: 1.34 ± 0.19 m/s; Females: i: 1.21 ± 0.21 m/s; ii: 1.22 ± 0.23 m/s
Sarramian et al. [92]	10 males & 8 females (16.0 ± 1.6 y)	National; within top 15 in their country (familiarized with the CA)	i) 30 min in-water WU (different speeds, leg-kick drills, short sprints, and a cool down)	15 min	ii) 15 min in-water warm-up + 1 × 3ML Pull-up; iii) 15 min in-water warm-up + 1 × 5 Weighted box jump; iv) ii + iii	4, 8 or 12 min (ind applied)	The inclusion of the pull-up and weighted box jumps did not elicit improvements compared to in-water WU. Different results were obtained between sexes T50m-males: i: 27.51 ± 1.06 s; ii: 28.01 ± 1.05 s; iii: 27.72 ± 1.04 s; iv: 27.49 ± 1.12 s; T50m-females: i: 30.87 ± 1.25 s; ii: 31.05 ± 1.00 s; iii: 31.05 ± 1.48 s; iv: 31.12 ± 1.27 s
Waddingham et al. [40]	8 males & 3 females (19.0 ± 1.2 y)	National	i) Dynamic mobility of the lower limbs (5 min) + 400 swim, 4 × 50kick/drill, 4 × 50 Freestyle (1 build, 2–25-m fast/25-m easy, 3-easy, 4-pace), 2 × 15-m Starts (all-out)	30 min	ii) i + 3 × 3 Band resisted squat; iii) i + 3 × 3 Weighted Jump Squat (15% bodyweight); iv) i + 2 × 5 Drop Jumps	ii) 6 min iii)3 min iv) 15 s	To improve the swim start performance, resisted band squats can be included in a race timeline alongside in-water WU T15m: i: 6.81 ± 0.42 s; ii: 6.70 ± 0.46 s (*d* = 0.30); iii: 6.86 ± 0.42 s (*d* = 0.40); iv: 6.84 ± 0.44 s (*d* = 0.04); increases in peak power of 6.9%, 7.8% and 2.9% were observed in dryland tests during i, ii and iii, following 6 min, 3 min and 15 s

*y* Years old, *WU* warm-up, *WR* World Record, T5–50 m: Time performed in 5–50 m swimming, *CA* Conditioning activity, *d* = Cohen’s d (effect size), *La*^−^ Blood lactate concentration. *PAP* Post-activation potentiation, *PAPE* Post-activation performance enhancements, *SR* Stroke rate, *SL* Stroke length, *RPE* Rate of perceived exertion effort, *UUS* Underwater undulatory swimming, *ML* Maximal Load, *DD* Dive distance; *FT* Flight time, *VxH* Horizontal velocity of the hip during flight, *BT* Block time, *DV* Dive velocity, *ISP* Isolated swimming phase, *RFD* Rate of force development, *BW* Body weight, *DJ* Drop Jumps, *CMJ* Countermovement jump, *eRFD* eccentric rate of force development, *IES* Index of explosive strength

##### Effects on the Swimming Start Performance

Two different studies conducted by our group compared a 400-m in-water warm-up followed by a dynamic lower limbs stretching protocol (i), with the same warm-up followed by ii) 3 lunges at 85% of one repetition maximum (RM), or iii) 4 squats on an eccentric flywheel device [53, 85]. After 8 min of rest, both studies obtained improvements on the dive distance, flight velocity, and time at 5 m, although these improvements were more significant after the protocol including the eccentric flywheel device (∆ = 7.2% vs ∆ = 14.5%). In a subsequent study by Cuenca-Fernández et al. [86], the same in-water and flywheel warm-up was studied with participants being tested on a swimming block equipped with force plates. The results of this study [86] showed improvements in mean and peak vertical forces, which resulted in an improvement of the resultant take-off velocity which may be due to a greater stimulation in the front leg by the eccentric overload [33]. Two recent studies obtained similar results but without using sophisticated equipment [40, 87]. In the study of Waddingham et al. [40], a 400-m varied pace in-water warm-up that included 2 × 15-m maximal swim starts, was compared to the same warm-up followed by: i) 3 × 3 resistance bands squat protocol (loads between 27.2 – 68 kg; ii) 3 × 3 weighted jump squats (15% bodyweight), and; iii) 2 × 5 drop jumps from a box [40]. Interestingly, the 15-m time improved after only the resistance bands squat protocol, which led the authors to conclude that this could be a simple way to provide lower limbs stimulation during the transition phase. In the study of Ðurovic et al. [87] a 1,600 m in-water warm-up that included 4 × 50-m front crawl (15-m maximal swim starts), was compared to the same warm-up followed by 3 × 5 drop jumps from a box (0.40 m). After 8 min of rest, the experimental protocol improved performance at the swim start to 15 m (∆ = 2.31%), and there were improvements in the eccentric RFD (∆ = 12.83%) and the index of explosive strength (∆ = 12.43%) on a countermovement jump (CMJ) test. However, other performance variables such as CMJ-height or CMJ-power did not significantly improve following experimental protocols compared to the control treatment. Hence, the authors argued that athletes with some experience in jumping probably needed more stimuli to increase muscle temperature, activation, and muscle–tendon stiffness to underpin the effects of PAPE on the CMJ tests.

Two studies analyzed the 15 m swim start section of a full 100-m race after different ballistic exercises. McGowan et al. [47] evaluated the effects of medicine ball throw-downs and box jumps after a standardized 1350-m in-water warm-up and observed that swimmers obtained trends (*p* > 0.1; ∆ = − 0.72%) for better times after including dryland exercise. Interestingly, the core temperature decrease during the transition phase (15 min) was lower during the experimental condition when compared to control condition (− 0.24 ± 0.13 vs − 0.64 ± 0.16 °C). Therefore, McGowan et al. [47] concluded that the ballistic dryland circuit could play a "reactivation" role after the standardized warm-up. More recently, de Arruda et al. [88] compared a standard 30-min in-water warm-up with a 15-min in-water warm-up followed by: i) loaded lunges (3 × 85% RM), or; ii) three pull-ups and box jumps (1 × 5 [10% body weight]), and; iii) a combination of the first and second protocols [i.e., complex-training]. After 4, 8 or 12 min of rest (individually applied), swimmers exhibited greater dive distances after all the experimental warm-ups thus improving the time over the first 5 m; however, they achieved lower values for horizontal hip velocity [88]. Importantly, the complex training protocol which combines both high load and ballistic exercises, exhibited the better improvements in 15-m time (i: 7.58 ± 0.50 s; iii: 7.69 ± 0.47 s; iv: 7.53 ± 0.47 s). Thus, such activities could potentially influence the underwater phase.

###### Summary

The evidence suggests that high-loaded CAs following the in-water warm-up would have higher influence than ballistic CAs on improving both the kinetic and the kinematic variables of the swim start performance. In any case, the warm-ups including jumping or plyometric exercises can be quite effective even with low dosage application. Thus, both approaches could be recommended to keep the body temperature.

##### Effects on Flutter Kicking and Underwater Undulatory Swimming

During the last years, the effects of PAPE have been studied on specific components of overall swimming such as the flutter kick and the underwater undulatory swimming (UUS). Ng et al. [89] compared the effects of a 1,400-m in-water freestyle warm-up that included different combinations of flutter kick drills, with the same type of warm-up over 700 m followed by 2 × 5 countermovement jumps (CMJ). After 8 min of rest, participants performed a 25-m maximal flutter kick effort exhibiting higher velocity, and kick frequency and thrust during the experimental condition. The authors [89] suggested that using only the participants' body weight during CMJ was a simple and effective way to acutely improve flutter kick performance. However, the different volumes between conditions may have influenced the results. More recently, Crespo et al. [90] compared a 600-m warm-up that included 3 × 10 m of maximal UUS, with the same warm-up followed by 1 × 4 squats on a flywheel in competitive swimmers. After 5 min of rest, the time to reach 10 m was faster for the condition including flywheel repetitions for both males and females, but only trends of improvement were obtained for the remaining UUS kinematic variables. These authors [90] argued that the improvements might have been greater with a longer rest interval between the CA and the test as participants reported a low competitive level of performance (FINA points < 500). In a recent study from the same group [91], a similar in-water warm-up was applied in age-group swimmers, but the flywheel repetitions were replaced by a series of four high-speed tuck jumps in order to test a protocol that could be applied in competition. Despite trends of improvement in push-off velocity, the results showed no improvement in UUS performance and kinematics following the tuck jumps, and no specific PAPE responses modulated by sex or swimmers’ strength level were observed for this age group. Based on the results obtained by Ng et al. [89] using body-weight high-speed CAs, the authors argued that possibly a superior number of repetitions was required to elucidate PAPE responses in UUS with this type of CA.

###### Summary

Although more evidence is needed, it appears that it is necessary to apply either a high load or several rounds of jumping to generate effective PAPE responses in some specific lower limb actions such as the flutter kick or the UUS.

##### Specific Effects on Swimming Performance and Stroke Patterns

Sarramian et al. [92] noticed that most of the previous studies examining PAPE in upper body tasks focused on pushing actions (e.g. bench press throws) but, surprisingly, no study had focused on PAPE after pulling actions. Thus, these authors [92] compared the effects of a 30 min in-water warm-up on a 50-m front crawl race with a 15 min in-water warm-up followed by: i) 1 × 3 maximal pull-ups, or; ii) the pull-ups followed by 1 × 5 box jumps (15% body weight). After 4, 8 or 12 min (individually applied), only males had a positive influence on the final time after the second protocol. The authors [92] argued that the pull-up exercise may not be an appropriate stimulus as it does not replicate the kinematic characteristics of the freestyle stroke. In two more recent studies [34, 53], an in-water warm-up of 400 m was compared to the same warm-up followed by 1 × 3 loaded lunges and pull-over (85% RM) exercises. After 6 min of rest, Cuenca-Fernández et al. [53] observed performance deteriorations in 50-m time after the loaded repetitions, while Cuenca-Fernández et al. [34] found increases in the rate of force development (RFD) and stroke rate, but with deteriorations in other kinetic and kinematic variables of swimming, such as velocity, stroke length, and acceleration, after the protocol including loaded repetitions. The lack of effects in both studies was suggested to be related to the high-resistance stimulus performed at low speed which might have an attenuating effect on neural output and subsequent swimming exercises [75, 93]. Meanwhile, although the increase in stroke rate in the study by Cuenca-Fernández et al. [34] could reflect some PAPE responses, the negative changes in stroke biomechanics could critically reduce lateral and sculling movements in the arm trajectory, thus producing a slippery effect in the stroke cycle with a reduction on the propulsive impulse [94].

Several studies compared the effects of in-water warm-ups (~ 1350 ± 390 m) followed by brief bouts of dry land exercises including ballistic exercises such as medicine ball throws, explosive jumps, burpees, push-ups and core exercises. Through this strategy, McGowan et al. [47] compared the effects of an in-water 1,350-m warm-up with the same warm-up followed by 5 min of dryland full-body power exercises, obtaining better performance in a 100-m freestyle race after 15 min of rest, and higher skin temperature when compared to the group that included the in-water warm-up only. Again, these authors [47] suggested that the combination of in-water warm-up and dry land exercises was a valuable strategy for maintaining elevated pre-competition core and muscle temperature, thus improving sprint swimming performance [2]. Subsequently, Dalamitros et al. [45], compared the effects of an in-water warm-up, with the same warm-up followed by: i) 2 × 3 repetitions of ballistic exercises (med ball throw downs, box jumps and crunches), or by ii) dynamic whole body stretching exercises with a work to rest ratio of 10:10 s. After 10 min of rest, the results in 50-m front crawl showed improvements in men after the protocol including dryland exercises. By contrast, women performed better after the protocol including dynamic stretching exercises. Thus, these authors [45] hypothesized that sex-related differences in factors such as muscle mass and flexibility [95] would explain the different effects of PAPE responses. On the other hand, Abbes et al. [56], tested the combination of an in-water 1200 m warm-up followed by different dryland exercises: i) 30-s maximal push-ups; ii) 30-s squat jumps, and iii) 30-s burpees. However, no significant differences were found in 50-m time after 10 min of rest therefore these authors [56] raised the possible interference of the age of their group (13.0 ± 2.0 years) on attaining PAPE responses. Testing trained and untrained participants, Dalamitros et al. [96], compared the effects of an in-water warm-up of 1100 m with a reduced warm-up of 600 m followed by 15 min rest and 1 × 5 loaded box jumps (at 10% of body weight). After 4, 8 or 12 min (individually applied), the results were slightly better in 50-m breaststroke in both groups after the PAPE condition, specifically at 25-m time (1.98 and 1.66% for trained and untrained, respectively), possibly due to improvements in the start phase. Therefore, the authors concluded that the PAPE responses were not influenced by the different competitive level of the participants. More recently, de Arruda et al. [88], compared a 30 min in-water warm-up, with a 15 min warm-up followed by 1 × 3 pull-ups and 1 × 5 box jumps (10% body weight). After 4, 8 or 12 min of rest (individually applied), the swimmers exhibited worse times in 50-m front crawl during the experimental condition, with superior values of stroke rate, and very similar values of stroke length compared to the control condition. These authors [88] concluded that the CAs were not efficient for performance improvement in the 50-m freestyle possibly because they resulted in residual peripheral fatigue.

Other authors have used elastic bands in an attempt to provide an easy-to-use exercise during warming up. For instance, Juarez et al. [83], compared the differences of an in-water warm-up of 1,200 m (10% at high-intensity), with the same warm-up followed by 4 sets (10 s) of pull-over repetitions with elastic bands in 25-m front crawl. The results were worse for the elastic band condition; however, performance was assessed 30 s after each band trial, limiting the possible PAPE effects. More recently, Barbosa et al. [97] compared a 1,400 m in-water warm-up with a reduced 700 m warm-up followed by 2 × 5 arm-pulls with resistance bands, obtaining improvements in swimming speed and thrust for the experimental group after 8 min of rest. These authors [97] concluded that warm-ups that included these conditioning sets would allow for greater muscle activation because of a greater time under tension [98], which could result in large PAPE responses. In this regard, despite the small effect on performance, a 2.5–3.0% improvement would translate into a meaningful reduction of 0.98–1.25 s in final race time.

Finally, other authors have attempted PAPE responses including distinctive dryland exercises. For instance, Nepocatych et al. [23], compared the effects of an in-water warm-up of 500-yards, with an experimental warm-up of 100-yards followed by 5 × 1 min arm pull exercises on a swim bench including upper body vibration at 22 Hz. After 3 min of rest, the experimental group obtained lower 50-yard times and HR. Meanwhile, Ramos-Campo et al. [15], tested an in-water warm-up of 1000 m, followed by 30 min of rest with the same warm-up followed by 10 min of rest and 5 min of dryland exercises, both in hypoxia or in normoxia. The results were better in 100-m front crawl after 5 min of rest for the warm-ups performed in hypoxia, especially with the inclusion of dryland exercises. These authors [15] highlighted that, although the dryland exercises only used body weight, the fact that they were performed in hypoxia could increase the stress of the load, obtaining positive effects in the subsequent exercise. In any case, higher body temperature and lower oxygen saturation were obtained in the warm-ups performed in hypoxia, thus possibly the limited oxygen availability would induce vasodilation to increase blood flow and oxygen delivery [11], which would contribute to the increase in body temperature and muscle $\dot{\text{V}}{\mathrm{O}}_{2}$ kinetics of subsequent exercise [99].

###### Summary

Applying maximal load CAs after the in-water warm-up does not appear to be as effective in improving swimming performance compared to the effects obtained in other sub-components of the race such as the swim start or the underwater phase; however, if there is a prolonged time between the cessation of the warm-up in the pool and competition, performing specific land-based exercises to maintain core temperature may be one way to improve performance during competition. Specifically, it seems that in-water warm-up of 1000–1200m followed by various sets of full-body ballistic CAs such as med ball throws, resisted bands, and explosive jumps for no more than 5 min, and with no more than 10–15 min of transition phase, could be effective in improving swimming performance. It is important to mention that the size of the effects is mostly low, and sometimes enhancements are not obtained in all participants. Still, these small changes could be especially relevant in sprint events.

#### Combined Warm-Ups Including External Heat Elements

Some studies have tested routines focused on maintaining muscle temperature after the warm-up (Table 5). De Vries [12] first tried hot showers as a method of passive warm-up, but they obtained deteriorations in performance compared to the in-water warm-up. Adams & Psycharakis [5], did not obtain differences in 100-m performance between an active in-water warm-up, a passive warm-up in a sauna, and a mixed warm-up including 10 min of each protocol. Nevertheless, it has been reported that passive warm-up could have a greater ergogenic effect on short-term dynamic performance (< 5 min) at faster contraction speeds [1]. Another three studies tested an in-water warm-up (~ 1300 m) combined with dryland exercises wearing or not heated jackets. In McGowan et al. [44], there were no differences between any of the conditions in 100-m breaststroke times after 15 min of rest. On the contrary, in McGowan et al. [47] and McGowan et al. [100], the swimmers obtained better values in the 100-m front crawl time for the warm-up including dryland exercises wearing heated jackets, obtaining an increased local upper body hemoglobin concentration. In the three studies [44, 47, 100], pre-test [La-] and HR values showed no differences, but the pre-time trial skin temperature was higher for the combined warm-ups including heating elements. Thus, these authors [44, 47, 100] concluded that the combination of a traditional pool warm-up followed by dryland exercise circuit completed alone, or including passive heat, could trigger relevant physiological responses leading to performance enhancements in real-world competition settings. Later, Wilkins and Havenith [13], compared in a 50-m front crawl a 1,600-m warm-up followed by 1 × 4 plyometric push-ups with the same warm-up using heated jacket elements during the subsequent passive recovery. Performance results were slightly better for the group including heated jackets at 25 and 50-m mark, with higher stroke rate and stroke count. Thus, these authors [13] attributed these improvements to a greater preservation of muscle temperature between warm-up and performance.

**Table 5:** Combined warm-up including external heat elements (*n* = 6)

References	Participants, Sex & Age	Level & Experience	Control condition	Rest	Experimental condition	Rest	Main findings & results
Adams & Psycharakis [5]	8 males (20.1 ± 1.8 y)	Competitive	i) 20 min in-water WU including a freestyle base set, preferred stroke (containing kick, pull, and drills), start and turns, before finishing with a 200 m swim	20 min	ii) No warm-up: sit in a sauna for 20 min iii) Mixed warm-up: 10 min in-water warm-up and 10 min sauna	20 min	The active and mixed WU conditions were most effective on 100-m individual’s preferred stroke T100m: i: 61.1 ± 6.4 s; ii: 62.2 ± 5.7 s; iii: 61.4 ± 6.1 s. The HR and RPE were lower after the active warm-up
De Vries [12]	13 males	Competitive	i) swimming (500 yards slowly and continuously)	No rest	ii) hot shower (6 min)	No rest	The warming up by 6 min hot showers had no positive effects
McGowan et al. [44]	6 males & 4 females (20 ± 1 y)	National and international (males: 800 ± 86 FINA points) (females: 813 ± 126 FINA points)	i) Standardized in-water WU of 1,350-m (400-m Freestyle [< 50% HRmax]; 4 × 100 m freestyle [60%HRmax]; 4 × 50 m breaststroke (60%HRmax) (drill; 25-m high; 25-m easy); 100-m freestyle; 2 × 50-m freestyle, personal best + 3-s (hand paddles); 2 × 25-m dive breaststroke (95% HR); 100-m Freestyle (50% HRmax)	30 min seated with the only activity to change into their racing swimsuit	ii) i + 5 min dry-land-based exercise routine [2 × (3 × medicine ball throw downs (2 kg), 3 × 10 s simulated butterfly kicks and 3 × tuck jumps)] wearing heated tracksuit pants with integrated heating elements over the backside and knee	15 min	Sprint 100-m breaststroke start, turn, and finish times were not enhanced after ii compared to i, despite eliciting higher skin temperature immediately before test initiation T100m: i: 68.6 ± 4.0 s; ii: 68.4 ± 3.9 s (*d* = -0.05); T15m: i: 7.3 ± 0.6 s; ii: 7.3 ± 0.6 s (*d* = 0.02); lactate pre time trial: i: 1.4 ± 0.6 mM/L; ii: 1.2 ± 0.3 mM/L (*d* = -0.35); HR pre time trial: i: 83 ± 18 bpm; ii: 84 ± 14 bpm (*d* = 0.68); upper body skin temperature pre time trial: i: 31.9 ± 1.4 ºC; ii: 31.1 ± 3.1 ºC; lower body skin temperature: i: 30.0 ± 1.6 ºC; ii: 29.2 ± 1.5ºC
McGowan et al. [47]	11 males & 5 females (16 ± 1 y)	National junior (100 m time: 59.41 ± 3.48 s)	i) Standardized in-water WU (25 min) of 1,300-m (400-m Freestyle (easy); 3 × 100-m medley (100-m: kick, drill, swim); 3 × 100 freestyle (80,90,95% race-pace); 4 × 50-m (15-m race pace, 35-m easy); 4 × 25 m Freestyle (dive start, race pace)	30 min seated with the only activity to change into their racing swimsuit	ii) i + wearing heating elements (Passive); iii) i + 5 min dry-land-based exercise routine [2 × (3 × medicine ball throw downs (2 kg), 3 × 10 s simulated butterfly kicks and 3 × 0,4 m box jumps)] (Dry-land) iv) ii + iii (Combo)	15 min	An improvement in 100-m freestyle time-trial performance was demonstrated when dryland-based activation exercises were completed alone (∼0.7%), and in combination with the wearing of a heated tracksuit jacket (∼1.1%). A smaller decline in core temperature during transition was strongly associated with faster time-trial performance T100m: i: 60.7 ± 3.36 s; ii: 60.37 ± 3.15 s (*d* 0.12); iii: 60.26 ± 3.50 s (*d* = 0.18); iv: 59.9 ± 3.7 s (*d* = -0.27); T15m: i: 7.23 ± 0.17 s; ii: 7.03 ± 0.24 s (d = 0.45); iii: 7.13 ± 0.16 s (*d* = -0.05); iv: 6.86 ± 0.19 s (*d* = 0.92); skin temperature: pre time trial: i: 33.1 ± 0.3 ºC; ii: 33.9 ± 0.3 ºC; iii: 33.3 ± 0.3 ºC; iv: 34.3 ± 0.1 ºC; no differences in La^−^ and HR;
McGowan et al. [100]	12 males (20 ± 3 y) 13 females (20 ± 2 y)	International and national-level (males: 50,8 ± 1,8 s and 791 ± 76 FINA points) (females: 55.6 ± 1.2 s and 824 ± 56 FINA points)	i) Standardized in-water WU of 1,350-m (400-m Freestyle (< 50% HRmax); 4 × 100 m Freesyle (60%HRmax); 4 × 50-m Freestyle (60%HRmax) (drill; 25-m high; 25-m easy); 100-m Freestyle; 2 × 50 freestyle, personal best + 3 s (hand paddles); 2 × 25-m dive Freestyle (95% HR); 100-m Freestyle (50% HRmax)	30 min seated with the only activity to change into their racing swimsuit	ii) i + 5 min dry-land-based exercise routine [2 × (3 × medicine ball throw downs (2 kg), 3 × 10 s simulated butterfly kicks and 3 × tuck jumps)] wearing heated tracksuit pants with integrated heating elements over the backside and knee	15 min	Combining an in-water WU with the use of heated jackets and dryland activation exercises during the transition phase (15 min) can yield up to a 0.8% or a 0.4 s improvement over the 100 m-freestyle event. Improved maintenance of core temperature in the transition phase as well as augmented local upper-body hemoglobin concentration appeared as key mechanisms contributing to the improvements in overall sprint freestyle performance T100m: Males: i: 53.7 ± 2.0 s; ii: 53.2 ± 1.5 s; Females: i: 58.9 ± 2.2 s; ii: 58.4 ± 2.0 s; T15m: i: 6.2 ± 0.3 s; ii: 6.1 ± 0.3 s; Females: i: 7.1 ± 0.4 s; ii: 6.9 ± 0.4 s; Turn times were not different between conditions (p = 0.08); Lower body peak impulse was similar between conditions. Lactate before time trial: i: 1.4 ± 0.7 mM/L; ii: 1.4 ± 0.8 (d = -0.07); HR before time trial: i: 82 ± 14 bpm; ii: 83 ± 15 bpm (*d* = 0.10); upper body skin temperature before time trial: i: 30.2 ± 2.0 ºC; ii: 30.1 ± 2.4 ºC (*d* = -0.04); lower body skin temperature before time trial: i: 29.4 ± 1.2 ºC; ii: 29.4 ± 1.5 ºC (*d* = 0,05); RPE increased in ii (*d* = 0.77); Upper and whole-body thermal sensation increased toward feeling warm (+ 2) in ii (*d* = 0.61 – 1.18); Total hemoglobin concentration: i: 30 ± 18; ii: 81 ± 25 (*d* = 1.45)
Wilkins et al. [13]	12 males (21 ± 1.8 y) 4 females (20 ± 1.7 y)	Elite; FINA points 651 ± 10; T50m: males: 23.83 ± 0.76; females: 27.15 ± 0.66 (16.7 h/week), 13.3 ± 2.7 years of experience	i) in-water warm-up of 1,600-m as: 400-m freestyle; 200-m Pull; 200-m Kick; 200-m Drill (Fins), 200-m Individual Medley, 4 × 50-m freestyle: 1) Push 15-m underwater fly kick; 2) 15-m spin drill; 3) dive 15-m race pace; 4) dive 25-m race pace, 200-m easy) + 4 plyometric press-ups	30 min seated (without heated jacket)	ii) i + wearing heat jackets during the rest	30 min seated with heated jacket	A 30-min period of upper body external heating post-warm-up leads to a significant improvement in 25 m sprint swimming performance, upper body force and power output T25m: i: 11.84 ± 1.0 s; ii: 11.72 ± 1.0 s; 50 m: i: 26.51 ± 2.0 s; ii: 26.30 ± 2.1 s; SR: i: 53 ± 2.9 cyc/min; ii: 55 ± 3.7 cyc/min; stroke count: i: 42 ± 4.5; ii: 44 ± 5.0; starting strength in push-up and peak force were greater in ii by 10.1% (*p* < 0,05) and 10.7% (*p* = 0,097). No differences in HR and RPE

*y* Years old, *PAP* Post-activation potentiation, *WU* Warm-up, *WR* T25–50–100 m: Time performed in 25–50–100 m swimming, *CA* Conditioning activity, *d* = Cohen’s d (effect size), La^−^: Blood lactate concentration, *SR* Stroke rate, *SL* Stroke length, *HR* Heart rate, *RPE* Rate of perceived exertion effort, *RFD* Rate of force development

##### Summary

Previous research has shown that, the use of clothing including heating elements during dryland circuits, or simply during a 20–30 min transition phase, could improve swimmers' performance. Although the improvements are sometimes very small, they represent a margin that can and often decides sprint races.

#### Only Dryland Activities

Since swimming is developed in the aquatic environment, one aspect that has also attracted the interest of researchers is whether it is possible to obtain the same warm-up effects through dryland exercises (Table 6). De Vries [12] compared a 500-yards in-water warm-up, with full body calisthenics circuit, and massage (for 10 min). Interestingly, only the in-water warm-up was effective in reducing the 100-yards post-test time. A few decades later, Bobo [66] found no differences in 100-yards time between an in-water warm-up and bench press practice performed for 5 min, however, the 100-yards time performance was averaged in a set of 5 × 100 yards, which presumably entailed fatigue from the last sets. Nepocatych et al. [23], compared the effects of an in-water warm-up with a 5 × 1 min arm pull on a swim bench including upper body vibration at 22 Hz. After 3 min rest, the 50-yards time was similar in both conditions, and these authors [23] concluded that the lack of greater improvements after the dryland warm-up could be due to the inability to prepare the race-specific muscles as in-water warm-ups. Similarly, Kilduff et al. [101] tested a standard in-water warm-up of 1,700 m with a set of 1 × 3 squats at 87% RM. After 8 min of rest, higher peak vertical force and peak horizontal force were obtained after the dryland CAs, but no variations were obtained in 15-m time (Table 6). In this case, these authors [101] concluded that the dryland stimulus produced PAPE responses since a similar start time compared to the swimmer’s traditional race-specific warm-up was obtained.

**Table 6:** Only dryland warm-up (*n* = 8)

References	Participants, Sex & Age	Level & Experience	Control condition	Rest	Experimental condition	Rest	Main findings & results
Bobo et al. [66]	10 males	Competitive	i) bench press	5 min	ii) exercises in the water	5 min	There were no differences between conditions in a 5 × 100-yards set
Costa et al. [103]	13 males (22.7 ± 1.4 y)	Practitioners for at least 2 years and experts in muscle-stretching exercises	i) 50-m front-crawl pilot test	No reported	ii) static stretching (2 × 30 s [15 s rest]); iii) PNF (2 × 30 s [15 s rest]), both in quadriceps and pectoralis	No reported	The acute effects of stretching negatively impacted performance in 50-m freestyle T50m: i: 32.12 ± 2.92 s; ii: 32.92 ± 2.51 s; iii: 33.52 ± 3.07 s
De Vries [12]	13 males	Competitive	i) swimming (500-yards slowly and continuously)	No rest	ii) calisthenics (ribs, flexing the hips, and stretching the long back muscles for 25 reps; chest muscles, abdominals, hip joint flexors, and strengthening lower back muscles 15 reps; strengthening abdominals and hip joint flexors for 100 reps;	No rest	Warming-up by swimming 500-yards was effective in reducing the subsequent 100-yards time trial by a mean difference of 0.44 s. The warming up by calisthenics had no effects. The freestylers as a group showed a significant decrease in speed in their trials after calisthenics warm-up
Iizuca et al. [102]	9 males (20.2 ± 1.0 y)	Experienced, national-level	i) 10 min in-water WU including 2 starts	No reported	ii) Trunk stabilization a) elbow-knee (held for 60 s); b) elbow-knee with alternative arm raise (30 times); elbow-knee with alternative leg raise (30 times) (15 s in between)	No rest	Trunk stabilization exercises led to immediate improvements in swim start performance DD: i: 3.14 ± 0.31 m; ii: 3.19 ± 0.30 m (*d* = 0.16); FT: i: 0.42 ± 0.08 s; ii: 0.42 ± 0.08 s (*d* = 0.01); Entry time: i: 0.40 ± 0.08 s; ii: 0.38 ± 0.07 s (d = 0.26); T5m: i: 0.82 ± 0.03; ii: 0.81 ± 0.03 s (*d* = 0.52); Entry velocity: i: 5.28 ± 0.20; ii: 5.27 ± 0.27 (*d* = 0.06); V5m: i: 4.61 ± 0.46 m/s; ii: 4.87 ± 0.35 m/s (*d* = 0.63). The rate of speed reduction decreased by 5.17% (*p* = 0.03)
Kafkas et al. [105]	14 females (22.5 ± 2.5 y)	Sub-elite; 5 years of experience (16 h/week)	i) 5 min run	3 min	ii) i + stretching 2 × 30 s (shoulder extensor, shoulder flexor, pectoralis, latissimus dorsi, adductor, hurdlers, hip rotator, bent-over toe raise, quadriceps and calf; iii) i + 1,200-m warm-up (400-m moderate swim; 4 × 50 leg kicks, 4 × 50-m drills, 4 × 50-m built, 25-m sprint and 150-m easy); iv) i + 10 min of Dry-land exercises over a 13 m distance: High-knee walk; Straight-leg march; Hand walk; Lunge walks; Backward lunge; High-knee skip; Lateral shuffle; Back pedal; Heel-ups; High-knee run	5 min	The best 50-m front crawl and breaststroke was found after in-water WU. Some positive responses to dryland WU revealed the swimmers’ individuality and confirmed the idea that warm-up procedures should be considered as an individualized approach to optimizing swimmer performance Crawl: T50m: i) 29.8 ± 2.3 s; ii) 30.7 ± 2.2 s; iii) 28.0 ± 2.9 s; iv) 28.4 ± 3.1 s; HR_Pre50: i) 88 ± 8 bpm; ii) 91 ± 9 bpm; iii) 105 ± 11 bpm; iv) 103 ± 12 bpm; RPE: i) 15 ± 2; ii) 15 ± 2; iii) 15 ± 2; iv) 15 ± 2; Breaststroke: T50m: i) 41.5 ± 2.9 s; ii) 41.8 ± 2.1 s; iii) 39.7 ± 2.6 s; iv) 40.5 ± 2.7 s; HR_Pre50: i) 91 ± 8 bpm; ii) 93 ± 10 bpm; iii) 109 ± 13 bpm; iv) 107 ± 12 bpm; RPE: i) 15 ± 1; ii) 15 ± 2; iii) 15 ± 1; iv) 15 ± 1
Kilduff et al. [29]	7 males and 2 Females (22 ± 2 y)	International sprinters (within 5% of the national record); engaged in a land-based conditioning program for at least 2 years. Training: 11 h/week with 3 h/week dry-land	i) standard in-water WU of 1,700-m (300 m-easy; 6 × 100-m Freestyle [3 pull; 3 kick]; 10 × 50-m freestyle swim [2 × (50 m as 25 fast/ 25 easy, 50-m lowest stroke count, 50-m build-up), 2 × 50 m at 200-m race-pace]; 100 loosen)	8 min	ii) 1 × 3 reps at 87% RM	8 min	The PAP stimulus produced a similar time to 15-m compared to traditional race-specific warm-up, indicating a potential role for PAP during sprint swimming No time variation at 15 m; peak vertical force: i: 1462 ± 280; ii: 1518 ± 311 N; peak horizontal force: i: 770 ± 228 W; ii: 814 ± 263 N
Nepocatych et al. [23]	4 males (37 ± 10 y) 6 females (34 ± 8 y)	Master (Best 50-yd time: Males: 29,5 ± 7,0; Females: 26,3 ± 3,3); > 3 years of experience, ≥ 3practice/week	i) 500-yards in-water WU including at least 2 × 25-yards sprints at 90%	3 min	ii) 5 × 1 min upper body vibration (22 Hz) including arm pull on a swim bench	3 min	Swimmers did not perform better in 50-yards after acute upper body vibration 50 yard time: i: 29.1 ± 3.36 s; ii: 29.1 ± 3.55 s; RPE: i: 17 ± 2; ii: 16 ± 1; HR: i: 148 ± 15 bpm; ii: 139 ± 12 bpm; stroke-count: i: 35 ± 7; ii: 36 ± 6
Romney & Nethery [65]	10 males	Collegiate	i) No WU	No reported	ii) 1,000-m in-water WU iii) 15 min dryland warm-up	No reported	There were improvements in 100-yard time after the in-water WU: -0.75 s; and improvements after the dryland warm-up: -0.65 s; RPE and stroke count did not change

*y* Years old, *WU* Warm-up, *WR* T5–15–25–50–100 m: PNF: Proprioceptive neuromuscular facilitation, Time performed in 5–15-25–50–100 m swimming, *PAP* Post-activation potentiation, *d* = Cohen’s d (effect size); *SR* Stroke rate, *SL* Stroke length, *RPE* Rate of perceived effort, *ML* Maximal load, *DD*: Dive distance, *FT* Flight time, *BT* Block time

Iizuka et al. [102] compared a 10 min in-water warm-up including two swimming starts, with a protocol of trunk stabilization which included elbow-knee position (held for 60 s), elbow-knee position with alternative arm raise (30 times), and elbow-knee position with alternative leg raise (30 times). Immediately after these protocols, a swim start to 5 m was performed. The results were better after the trunk stabilization protocol for time and speed in 5 m. There were observable trends (*p* > 0.1) on entry time and distance in favor of the dryland protocol, without differences in flight time and speed of entry. These authors [102] suggested that deceleration due to water resistance at the moment of entry was important, so facilitation of the deep trunk muscles would increase body stability, and this would entail a reduction of entry time.

Costa et al. [103], compared the effects of two sets of stretching techniques applied on quadriceps and pectoralis (static stretching and proprioceptive neuromuscular facilitation [2 × 30 s]) on 50-m front crawl time. Although resting time was not provided, the results showed worse times for the stretching protocols compared to the non-activity condition. These authors [103] argued that stretching exercises may increase muscle compliance, which may limit the amount of cross-bridging and thus reduce the muscle ability to produce force [104]. Similarly, Kafkas et al. [105] compared: i) a 5-min run, with the same activity followed by: ii) a series of full-body stretching exercises (2 × 30 s); iii) a 1,200 m warm-up, and; iv) 10 min of lower limb exercises (e.g., high knee walks, lunge walks or heel-ups). After 5 min rest, the best results in 50-m front crawl and 50-m breaststroke were obtained for the in-water warm-up protocol followed by the running exercises. These authors [105] argued that, although static stretching is a widespread technique in warm-up routines, it may influence neural mechanisms that could negatively affect muscle performance during swimming by reducing motor unit activation and muscle–tendon unit stiffness [106]. However, it is important to mention that the effects of stretching might have been attenuated if a full warm-up had been applied afterwards [107].

##### Summary

Dryland warm-up exercises could be an effective alternative when a swimming pool is not available for the CAs. These activities are also capable of ensuring an increase in muscle temperature and HR, which guarantees the cardiovascular adaptations that modulate the improvement in performance. In any case, it is possible that additional specific neuromuscular adaptations may occur during warm-ups conducted in the water, which is why their implementation or combination with dryland activities is recommended.

### What are the Main Conclusions, Considerations and Gaps that Should be Addressed in Further Research?

#### Responders and Non-Responders

Certain subgroups of participants may obtain performance enhancements from the intervention, while others may see their performance impaired [27]. In some cases, this could be conditioned by the individual background of each subject in relation to their ability to tolerate the load before the test, or by the lack of equal rest, which could also be related to the latter [6, 20]. In other cases, the improvement in muscle force production verified by means of dryland exercises may have not always translated into performance improvements in the water, an aspect that has considerably limited the conclusions in favor of PAPE responses. In any case, it is important to mention that, when the mean response of a given sample or group denotes a lack of effect of an intervention protocol, this may be due to different responses from each individual, which, when pooled and averaged, resulted in an apparent lack of effect. Thus, it seems important to examine the role of intra-individual variation in stimulus response, as this would complement the data on group mean responses [108].

On the other hand, although potentiation responses have been reported to be more likely in highly trained athletes (*d* = 0.41) compared to their weaker counterparts (*d* = 0.32), due to their greater resistance to fatigue and fast fiber proportion [25], this theory has not been confirmed in swimming as only four studies have addressed this question, with only one obtaining relations in favor (*r* = 0.74) [57]; another one showing no relations [91]; and two others obtaining PAPE responses regardless of the training status of the athletes [83, 96]. Thus, as the PAPE effects have been mainly related to temperature rising mechanisms and fluid shifts [19], this would not match with the previous paradigm of responders and non-responders. In addition, a movement-specific relationship between the CAs and those completed in the subsequent task has always been considered one of the main modulators for the improvements in performance [44, 100]. However, improvements have also been obtained after some protocols where the CAs did not share any relation with the task, meaning that sufficient, rather than specific stimulation, would be enough to trigger performance enhancements [27, 34].

#### Sex and Age-Related Effects

Overall, 550 participants were included in this review, but only 21% of the sample consisted of women. Furthermore, only five of the studies included both sexes with the results being reported separately. Therefore, it is difficult to draw conclusions about possible sex-regulated effects on specific responses to the warm-up procedures as women are clearly under-represented. Since males tend to have larger cross-sectional area of type II twitch fibers and shorter contraction times than females [109], some studies have concluded that males may experience higher PAPE responses in short explosive tasks, while females may perform better in other longer tasks due to greater fatigue resistance than males [20, 110–112]. Of the studies included in this scoping review, Hancock et al. [59] did not obtain sex-related PAPE differences in 100-m performance in response to a resisted in-water swimming protocol. In contrast, Sarramian et al. [92] observed that only males had better performances after a combined in-water and dryland warm-up, attributing these improvements to the greater cross-sectional area of males’ type II fibers [109]. Similarly, Wilkins and Havenith [13] obtained performance enhancements in 50-m performance (∆ = 1.01%) only in males after a combined in-water and dryland warm-up wearing heated jackets. In this case, these authors [13] attributed these differences to the different thermal perception of females to innocuous heat [113]. Interestingly, Dalamitros et al. [45] obtained improvements in 50-m time in males after the protocol including dryland exercises (*d* = 0.29) and in females after the protocol including dynamic stretching exercises (*d* = 0.31), while Crespo et al. [90], observed improvements in UUS time to 10 m after the flywheel loading protocols, obtained in males from a higher push-off speed, and in females from a higher kicking frequency.

On the other hand, the age of the participants would be a relevant point in the PAPE responses as growth influences the level of strength, and thus the strength-differentiated responses [20, 114, 115]. In swimming, age and maturational state could be unrelated to experience in practice, as some practitioners begin the activity at certainly advanced ages and others at a very early age. This confounding factor should be taken into account when interpreting the results of swimming studies, as participants tend to have a low average age compared to other sports. In this scoping review there were no studies that tested whether similar CAs elicited different responses in samples of different ages. In any case, Abbes et al. [56], obtained non-significant improvements in a group of young swimmers (13.0 ± 2.0 years) after an in-water and dryland warm-up, while in a subsequent study, Abbes et al. [79], observed a significant performance deterioration in the same group of swimmers after a tethered swimming warm-up using hand-paddles. Therefore, from these two studies it appears that the CA, rather than the age of the swimmers was the differentiating factor in these PAPE responses.

#### Transfer of Dryland to PAPE Responses in the Water

In the study of Dalamitros et al. [45], three of the participants reported a feeling of "bad catch and pull" after the dryland protocol, while Cuenca-Fernández et al. [34], reported a possible interference in the "perseveration" effect after slow speed movements during the loading protocols [116]. Therefore, despite upper body strength in dryland conditions has been assessed and related to swimming performance [94, 117–119], it is well established that the pattern of force production and the way that swimmers use their limbs to generate thrust determine the effectiveness of swimming propulsion [120]. In fact, increases obtained on the arm-pull thrust may reflect an acute enhancement of the neuromuscular mechanism [97]; however, the arm-pull thrust does not essentially represent the effective propulsion generated by the body, but rather the increase of the force conveyed per stroke against the water [34]. Therefore, the technical aspects of swimming mechanics determine the extent to which increased power is transferred to increased swim speed [58, 120]. Furthermore, wrist flexion, elbow elevation, body rotation on the longitudinal axis and internal rotation of the lower limbs are critical points in swimming [121, 122], but they are not replicated in any of the more common dryland exercises used in the pool. Thus, in-water warm-up routines would probably be better for triggering PAPE responses in swimming, while the addition of dryland exercises should be seen as a means to maintain the positive effects provided by the in-water warm-up.

#### Different Responses on Different Sub-Components of the Race

The swimming start performance appears to be a subcomponent of the race capable of being enhanced beyond what would be achieved with an in-water warm-up by using different combinations of dryland exercises, such as loaded squats, eccentric devices, drop jumps and various plyometric sets. In the case of free swimming, however, these exercises seem to have less effect as the responses seem to be more dependent on specific stimuli obtained from the in-water warm-up (Tables 4 & 6). According to some authors, the effects of PAPE appear to be greater on maximal and fast voluntary contractions than on cyclic movements [25, 26]. Thus, if the protocols are attempted to optimize the start, faster 15-m times could be achieved, and this could have a relevant impact on overall race performance as up to 30% of the final time in short races can be attributed to the start [40, 123]. In any case, studies in other endurance modalities have shown that potentiation strategies can have an influence on pacing, without necessarily showing an improvement in performance in the final result [124, 125]. Still, an appropriate priming strategy for the swimming phase could compensate the lack of effects on the start. An example of this was observed by Neiva et al. [71], obtaining better 100-m times after a short and medium warm-up (compared to a long one) without significant differences at the start phase.

In the case of sprint swimming, most research has studied the potential effect of CAs using the front crawl technique, with only a few of them focused on the breaststroke [44, 45]. In contrast, nothing has yet been investigated in backstroke, butterfly or individual medley, and there is a paucity of research on the effects of experimental warm-ups over distances greater than 100 m, even though the present findings suggest that properly executed CAs can provide PAPE-related improvements that could persist for several minutes. Thus, future studies should test whether improvements could be obtained over longer distances.

#### Psychological Factors and Motivation

The warm-up period is recognized as an opportunity for athletes to prepare mentally for an upcoming event, providing them with time to concentrate on the task ahead [1, 2]. In this regard, typical mental preparation strategies include visualization, attentional focus, and elevation of preparatory anxiety or relaxation to reach the optimal level of pre-competition arousal [4]. Orlick & Partington [126], claimed that the use of pre-competition psychological routines was a characteristic of successful Olympic athletes. However, it is important to note that the psychological changes obtained from these mental strategies could enhance but also impair performance in athletes regardless of the warm-up used. Specifically in swimming, McGowan et al. [46] identified that the main psychological concerns faced by swimmers in competition occurred during the transition phase, as swimmers needed to be mentally alert and focused on the race plan but at the same time, serene, and that this was particularly complicated if there were delays in the competition schedule. Alternatively, Hays et al. [127], stated that athletes' confidence is positively affected by the interaction with the coach and teammates, and by how comfortable the athlete is with the environment, which includes, for instance, if the tests are performed in the same training pool as usual or if participants compete with other competitors, or against themselves. Also, while some athletes may feel low confidence after some of the warm-up protocols and may not be able to achieve a good performance due to lack of motivation [105], for others, these interventions may produce a better perception of "readiness", which would improve their performance simply by putting more effort into the tasks or tests. For instance, McGowan et al. [47] described that their participants could not be completely blind to the interventions, so these authors acknowledged that improvements in 100-m performance may have been influenced by the placebo or learning effect.

## Limitations

The main concern found in many of the experimental studies that have included biomechanically similar pre-competitive loading protocols is that they have attributed subsequent responses in voluntary activities to a false post-activation potentiation (PAP) effect. Although PAP and PAPE can be observed simultaneously in some cases [128], the use of the term PAP may not always be appropriate to frame the short-term responses that occur in voluntary sport tasks following a stimulation protocol [19, 24, 27], as this is a muscle-memory mechanism originated by the contraction-induced effects of the myosin light chain (MLC) phosphorylation verified only with the electrically evoked twitch interpolation technique [129]. On the other hand, many studies included small sample sizes (10–15 participants), with females mostly unrepresented. Meanwhile, most of the loads or recovery times were not individually adapted but rather were set as the average for the total group, therefore, this could pose relevant confounding factors due to participants’ different training backgrounds (e.g., if strength resistance routines are usually performed by only a proportion of the participants).

Regarding the methods, it is worth mentioning that studies do not usually test on more than one occasion to establish consistent responses after similar or different warm-ups and thus, establish individual and optimal procedures. In addition, some approaches have looked for the optimal time to obtain the performance enhancements by repeating the same test to participants in consecutive rest periods (e.g., 4, 8, 12 and 16 min). Thus, the test itself could provide a carry-over effect from the early periods (i.e., 4 and 8 min), to the later periods (i.e., 12 and 16 min). Meanwhile, many warm-up procedures have been presented; however, most of the studies that intended to improve swimming performance through upper limb CAs ruled out the possible influence of the lower limbs on the results. Furthermore, the kinetic variables of swimming (e.g., force and impulse) are rarely evaluated or only measured in tethered conditions (i.e., without displacement), while some physiological parameters such as the muscle temperature, oxygen saturation and hemoglobin concentration, together with other psychological factors such as the level of anxiety, the motivation, or the adaptation of the swimmer to the experimental context, are seldomly assessed and could be as important as other modulating factors traditionally highlighted in the literature. Thus, the biological or physiological effects prompted by the warm-up could be biased by an inadequate procedure to detect those changes.

## Conclusion

Swimmers could optimize performance from a warm-up that includes a moderate mileage of water exercise (~ 1000 m) performed at an intensity of ≤ 60% of maximum oxygen consumption, especially if this is followed by ~ 5 min of dryland activities and leaves no more than 10–15 min of rest during the transition phase, as this would keep muscle activity and body temperature elevated until the subsequent swim race. Although some of the procedures in this scoping review have shown positive results, the application of some CAs seems unfeasible in competition as very specific equipment is required while swimmers are waiting in the call room (e.g., eccentric flywheels, vibration devices, pulley systems, etc.). Therefore, to complement the in-water warm-up, swim coaches and scientists require the design of exercises that meet the objectives of the preparation, require minimal equipment, and can be easily completed in a confined space, e.g., through jumping drills, med ball throws or elastic bands exercises, as they have shown promising results as reported in swimming literature and other sport settings. All of these methods should be combined with means to maintain body temperature, such as clothing or heaters, and should be evaluated in an integrative assessment based on the effects prompted on the biomechanical, physiological and psychological variables. Following these recommendations while in the call room may give swimmers a competitive advantage.

## Data Availability

Data supporting this review are available from the corresponding author upon request.

## Abbreviations

$$\dot{\text{V}}{\mathrm{O}}_{2}$$: Oxygen consumption
H^+^: Hydrogen ion
HR: Heart rate
PAPE: Post-activation performance enhancement
CA: Conditioning activity
FINA: Federation internationale de natation
PCr: Phosphocreatine
RPE: Rate of perceived exertion
PRISMA—ScR: Preferred reporting items for systematic reviews and meta-analysis, with the extension for scoping reviews
RM: Repetition maximum
UUS: Underwater undulatory swimming
CMJ: Countermovement Jump
PAP: Post-activation potentiation
MLC: Myosin light-chain
WU: Warm-up
SR: Stroke rate
SL: Stroke length
*d*: Cohen’s d (effect size)
[La-]: Blood lactate concentration
WR: World record
RFD: Rate of force development
